# Global spatial assessment of *Aedes aegypti* and *Culex quinquefasciatus*: a scenario of Zika virus exposure

**DOI:** 10.1017/S0950268818003102

**Published:** 2018-11-26

**Authors:** Alberto J. Alaniz, Mario A. Carvajal, Antonella Bacigalupo, Pedro E. Cattan

**Affiliations:** 1 Centro de Estudios en Ecología Espacial y Medio Ambiente – Ecogeografía, Santiago, Chile; 2 Laboratorio de Ecología, Departamento de Ciencias Biológicas Animales, Facultad de Ciencias Veterinarias y Pecuarias, Universidad de Chile, Santiago, Chile

**Keywords:** Arbovirus, exposure level, mosquito, spatial epidemiology, ZIKV risk

## Abstract

Zika virus (ZIKV) is an arbovirus transmitted mainly by *Aedes aegypti* mosquitoes. Recent scientific evidence on *Culex quinquefasciatus* has suggested its potential as a vector for ZIKV, which may change the current risk zones. We aimed to quantify the world population potentially exposed to ZIKV in a spatially explicit way, considering the primary vector (*A. aegypti*) and the potential vector (*C. quinquefasciatus)*. Our model combined species distribution modelling of mosquito species with spatially explicit human population data to estimate ZIKV exposure risk. We estimated the potential global distribution of *C. quinquefasciatus* and estimated its potential interaction zones with *A. aegypti*. Then we evaluated the risk zones for ZIKV considering both vectors. Finally, we quantified and compared the people under risk associated with each vector by risk level, country and continent. We found that *C. quinquefasciatus* had a more temperate distribution until 42° in both hemispheres, while the risk involving *A. aegypti* is concentrated mainly in tropical latitudes until 35° in both hemispheres. Globally, 4.2 billion people are under risk associated with ZIKV. Around 2.6 billon people are under very high risk associated with *C. quinquefasciatus* and 1 billion people associated with *A. aegypti.* Several countries could be exposed to ZIKV, which emphasises the need to clarify the competence of *C. quinquefasciatus* as a potential vector as soon as possible. The models presented here represent a tool for risk management, public health planning, mosquito control and preventive actions, especially to focus efforts on the most affected areas.

## Introduction

Zika virus (ZIKV) is a member of the family Flaviviridae. This virus is dispersed mainly through dipteran vectors of the genus *Aedes*; *Aedes aegypti* is considered the main [1–3]. ZIKV has the potential to cause permanent effects in the fetus, which is infected by transplacental transmission when the pregnant mother is infected with the virus [4, 5]. During 2016 numerous cases of microcephaly were reported in Colombia and Brazil, associated with pregnant women infected by ZIKV in the 2015–2016 summer of the southern hemisphere [6, 7]. The control of this vector is difficult due to the reproductive characteristics of *A. aegypti*, which can lay hundreds of eggs in a short period of time, making it a serious threat for public and community health [8, 9].

The global risk level was estimated by Alaniz *et al*. [10], who reported that 2.26 billion people had high or very risk levels of ZIKV exposure, while Messina *et al*. [11] estimated 2.17 billion people at risk. Both models considered only the transmission associated with the main vector *A. aegypti*. However, recent studies have proposed that *Culex quinquefasciatus* is susceptible to carry ZIKV, representing a new potential threat as a possible vector of ZIKV [12–19]. Other studies have reported that species of the genus *Culex* shown that *Culex pipiens* is not competent to transmit ZIKV, hence the current scientific evidence on *C. quinquefasciatus* as a vector of ZIKV virus remains under debate [20–23]. However, the possibility that *C. quinquefasciatus* could represent a new vector could modify the areas of influence of ZIKV worldwide, mainly in countries where *Aedes* is not abundant or has recently arrived [14]. It is important to consider that *Culex* mosquitoes are much more abundant than *Aedes* in some areas (e.g. in South America) [24, 25]; their distribution range is different than that of *Aedes* mosquitoes and they have diurnal feeding habits. Specifically, *Culex* has a wider distribution range, reaching sub-tropical regions and is present in areas with low risk of ZIKV associated with *A. aegypti* [14, 25]. This dissimilar distribution range of *Culex* mosquitoes could introduce ZIKV to areas where the conditions are unsuitable for its main vector. This risk could be associated with the distance from the zones of co-occurrence of these vectors and can be modulated mainly by the dispersal of infected secondary vectors [14, 26].

We present a scenario of the potential risk of ZIKV transmission associated with the potential competence of *C. quinquefasciatus* as a ZIKV vector and we update the previous estimation of Alaniz *et al*. [10] for the primary vector *A. aegypti* in a spatially explicit way. In particular, we determine: (A) the world distribution of *C. quinquefasciatus* and its potential interaction zones with *A. aegypti*; (B) the risk of ZIKV considering the new secondary vector and an update of the risk estimation for the primary vector *A. aegypti*; (C) a spatially explicit comparison of the risk zones of each vector worldwide and (D) Quantification and comparison of the people at risk associated with each vector, according to risk level, country and continent.

## Materials and methods

### Identification of vector world distribution and interaction zones

We used Species Distribution Modelling (SDM) based on the Maximum Entropy algorithm with MaxEnt 3.3.3k software [27, 28] to predict the distribution ranges of both ZIKV vectors. MaxEnt uses two types of input data: occurrence points of the target organism and a set of environmental variables. The aim is to predict the level of environmental suitability for the species based on its ecological niche requirements [28]. The SDM prediction could be homologated to the potential abundance of an organism [29]. This method has proven to be useful and reliable in the modelling of infectious disease vectors [10, 30]. To model the distribution of *C. quinquefasciatus* we compiled 3865 occurrences worldwide from the Global Biodiversity Information Facility (GBIF) [30]; Integrated Digitised Biocollections (https://www.idigbio.org); SpeciesLink (http://www.splink.org.br); MosquitoMap [31]; INaturalist (https://www.inaturalist.org), entomological collections and scientific papers [32, 33] (Supplementary data, File S1). The environmental variables used were the bioclimatic layers of WorldClim project with 2.5 arc min spatial resolution worldwide (approximately 5 km×5 km cells), plus elevation data [34]. To reduce the spatial autocorrelation and the geographical bias of occurrences dataset, we applied a spatial rarefy function, maintaining points that were separated by at least 15 km [35]. To reduce collinearity of bioclimatic variables we generated a preliminary model with the complete set of variables (19 bioclimatic, plus elevation) with a 15-fold cross-validation technique, calculating the percentage contribution and permutation importance of each. Then we applied the Shapiro–Wilk test to assess the normality of the dataset and a correlation matrix expressed in a correlogram using the absolute correlation coefficient [36] (Supplementary data, Fig. S1) to exclude highly correlated variables. The variables with high importance in the preliminary model with a low correlation coefficient (less than ± 0.7) were selected. The final models were constructed with a 50-fold cross-validation technique, 95% confidence interval (Lower CI) and with the selected variables only. The contribution of each variable was estimated independently using the Maxent algorithm (Supplementary data, Fig. S2). The accuracy of the model was assessed through the Area Under the Curve of the receiver operating characteristic, which estimates the sensitivity and specificity by partitioning the dataset into a training and test dataset; the test dataset was not used in the model construction (independent validation) (Supplementary data, Fig. S3) [27]. The uncertainty corresponds to the standard deviation (s.d.) of the predicted suitability to each vector (Supplementary data, Figs S4 and S5). The importance of each variable was corroborated through a Partial Least Squares Regression in R open-source statistical language (Supplementary data, Fig. S6).

### ZIKV risk estimation: potential secondary vector and update on the primary vector

We quantified the risk associated with exposure to *C. quinquefasciatus* as ZIKV potential secondary vector by considering the following parameters: (A) potential interaction between vectors, considering the probability of co-occurrence of the primary vector (*A. aegypti*) with the potential vector (*C. quinquefasciatus*) and the potential dispersion of infected secondary vectors from interaction zones into non-interaction zones; (B) suitability or potential abundance of the secondary vector (Supplementary data, File S2); and (C) Human population density (Supplementary data, Fig. S7).

To determine the interaction zone between vectors, we overlapped the SDMs of *A. aegypti* [10] and *C. quinquefasciatus*, identifying where high suitability areas for both species coincide. Considering the recent studies on mosquito species, we hypothesize that it could be possible for *A. aegypti* to infect hosts with ZIKV and then the secondary vector could become infected by feeding on the same infected hosts. These common areas were identified by reclassifying the probability of the presence of each vector in four levels, converting the continuous probability grid from 0 to 1 into a new discrete grid with four categories. This method divides the range of probabilities into four levels 0–25% (null), 25–50% (low), 50–75% (medium), 75–100% (high) of the complete range of probabilities of the SDM. This could be considered a more parsimonious way to determine each one of the levels because the thresholds which divide each one of the levels are scaled in relation to the probability range of each SDM. These new discrete grids of suitability were multiplied, obtaining a grid with levels of potential spatial interaction from null to very high, associated with the spatial co-occurrence of both mosquitoes [37]. These areas were named ‘Interaction zones’ (Supplementary data, Fig. S8). Additionally, we generated a sensitivity analysis assessing two more thresholds to categorise the four above mentioned levels, by integrating the uncertainty associated with the SD of each SDM. This threshold consisted of two scenarios of equal interval classification (Equations 1 and 2) (Supplementary Data, Tables S1 and S2):
1
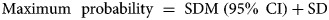

2
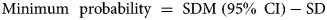

where SDM (95% CI) corresponds to the mean probability of presence estimated by the SDM.

To estimate the risk due to *C. quinquefasciatus*, we considered the previously calculated probability of co-occurrence as significant when the interaction levels ranked from medium to very high. The risk of ZIKV due to *C. quinquefasciatus* was estimated considering three factors: the distance from interaction zones, the probability of the presence of *C. quinquefasciatus* and the human population density. The distance from interaction zones was determined by considering a theoretical active dispersal distance of *C. quinquefasciatus* of 100 kms [38–41]. We generated a distance grid from the interaction zones, assigning four levels of proximity: high (from 0 to 100 km); medium (100–200 km); low (200–300 km) and null (>300 km). Then we reclassified this distance map, assigning a weight to each buffer (high = 3; medium = 2; low = 1; null = 0). This reclassified distance map was multiplied by the reclassified map of probability of the presence of *C. quinquefasciatus*, obtaining a grid with five levels from null to very high (0–5) (Supplementary data, Fig. S9).

To evaluate the risk of infection we used the human Population Density Grid (v4 of 2015) with 2.5 arc minute spatial resolution generated by the Socioeconomic Data and Application Centre of NASA [42]. To evaluate the population at risk, the population density grid was classified in four density levels: null (0–1 inhabitants/km^2^), low (>1–10 inhabitants/km^2^), medium (>10–100 inhabitants/km^2^) and high (more than 100 inhabitants/km^2^), assigning a value to each category (null = 0; low = 1; medium = 2; high = 3). Then this raster grid was multiplied by the grid developed in the previous steps (Equation 3), obtaining 11 levels, which were reclassified into five risk levels from null to very high (null 0; very low = 1–2; low = 3–4; medium = 6–8; high = 9–12; very high = 18–27) (see Supplementary Fig. S7).
3
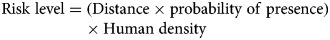


To update the ZIKV risk associated with *A. aegypti*, we applied the protocol of Alaniz *et al*. [10]. We used the same SDM previously reported, but updating the human Population Density Grid to the year 2015 [43].

### Spatially explicit comparison of the risk zones of each mosquito worldwide

To compare the risk zones, we used only the medium to very high levels of probability of presence. We reclassified each risk raster grid (*C. quinquefasciatus*) as a binary risk map. These maps were summed to generate a new map with three categories: (a) risk due to the presence of *A. aegypti* alone; (b) risk due to the presence of *C. quinquefasciatus* alone; and (c) risk due to the presence of both vectors (Supplementary data, Files S3, S4 and S5). Finally, we analysed the geographic distribution patterns of both vectors.

### Quantification and comparison of the people at risk associated with each mosquito

The risk was overlapped with a map of population count by a square kilometer of NASA. The product used was the Global Rural-Urban Mapping Project, Version 4 (GRUMPv4) [43]; this is an estimation of the human population in 2015 based on censuses. We quantified the population by risk level (from null to very high) and we estimated the percentage of the population potentially affected by country. This process was repeated for each vector, to describe the number of people at risk by each vector by country, continent and risk level.

## Results

### World distribution of *C. quinquefasciatus* and its potential interaction zones with *A. aegypti*

**Fig. 1.**
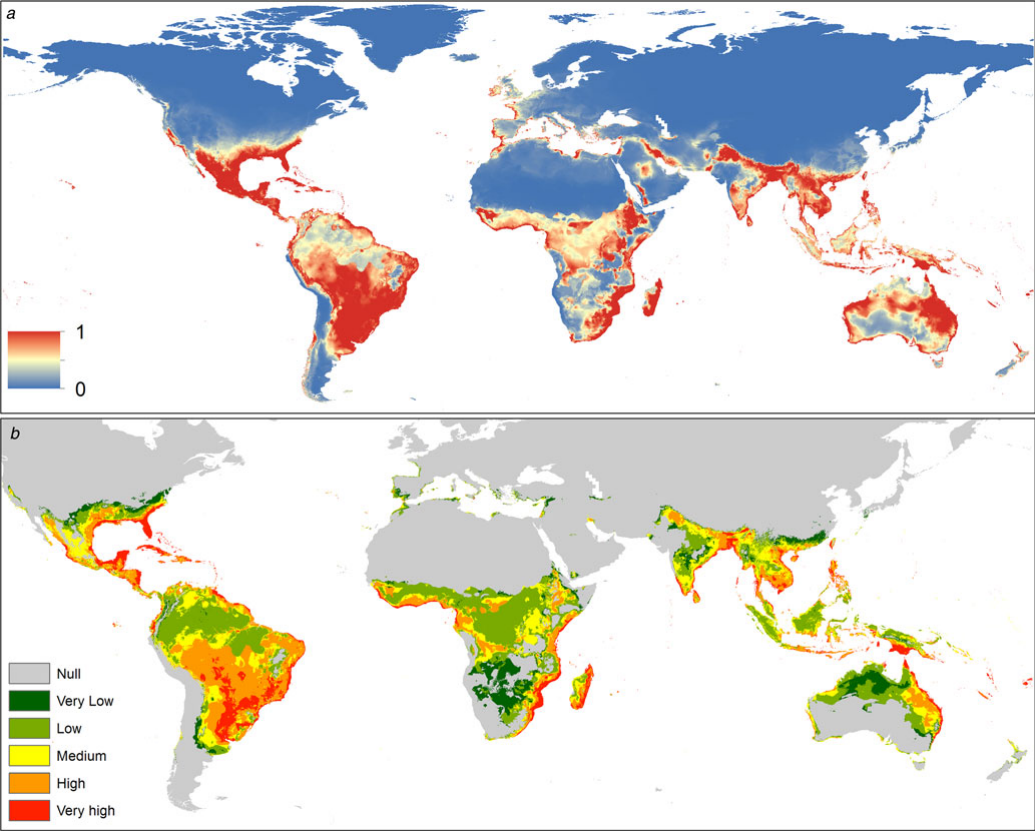
(a) Suitability map of *Culex quinquefasciatus* worldwide. The colours represent the suitability level from 0 (blue) to 1 (red). (b) Potential interaction zones between *Culex quinquefasciatus* and *Aedes aegypti* worldwide, categorised by levels of interaction.

The spatial distribution of *C. quinquefasciatus* spans from latitude 39° N to 39° S according to the model generated. In America it is present mostly in the Atlantic coast, however, there is a high probability of presence in Central America, Mexico, Chile and California (USA). In Africa, *C. quinquefasciatus* is present from latitude 10° N to the Cape of Good Hope in South Africa and there is a high probability of presence in the Mediterranean coast of Africa. In Asia, it is present from the Middle East to China, mainly in the Indian ocean coast. In Oceania, this mosquito is present in all countries. Finally, in Europe, it has a high probability of presence in the Mediterranean and Atlantic coasts (Fig. 1a).

The distribution of this mosquito seems to be limited by biogeographic barriers, including the Atacama Desert in South America, the Sahara Desert in Africa, the Himalayas in Asia and the deserts of south-central Australia.

The potential interaction with *A. aegypti* occurs mainly in tropical areas. In the Americas, the Caribbean Coast and southern Brazil have very high probabilities of potential interaction. In Africa the interaction is higher near the coast, decreasing inside the continent. The main interaction zones in Asia are in coastal areas. In Oceania, the Pacific coasts of Australia and Papua New Guinea have a high probability of interaction between these vectors. In Europe interaction occurs in southern Spain and Portugal on the Atlantic coast, while in the Mediterranean coast there is a medium level of potential interaction (Fig. 1b).

The uncertainty effect on the probability of presence levels and the interaction zones for both vectors was low, showing less than a ~1% of change on the estimated areas (Supplementary data, Table S1 and S2).

### ZIKV risk estimate by the new secondary vector and update on the risk of the primary vector

**Fig. 2.**
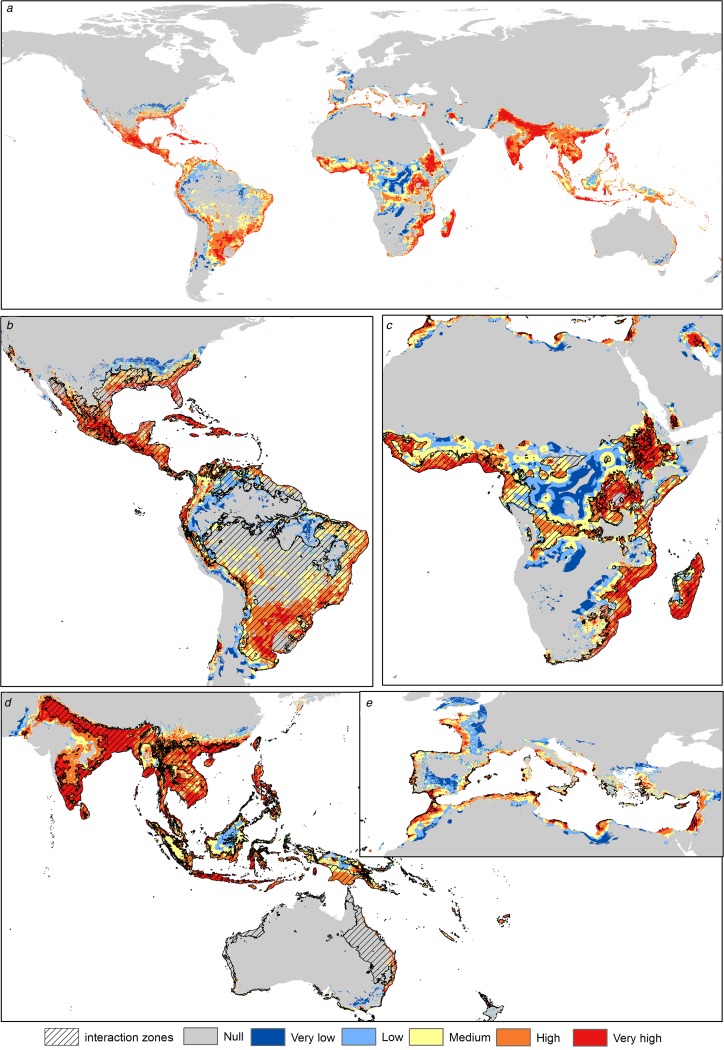
Transmission risk model of ZIKV due to the vector *Culex quinquefasciatus*. (a) Map of the transmission risk of ZIKV worldwide by *C. quinquefasciatus*. (b) Zoom to the transmission risk map of America. (c) Zoom to the transmission risk map of Africa. (d) Zoom to the areas with higher transmission risk in Oceania. (e) Zoom to the transmission risk map of Europe.

Asia shows the highest risk levels for *C. quinquefasciatus*, mainly in India, China and Thailand. The risk areas in America are concentrated in Central America and the Atlantic coast of South America; however, Mexico and the USA have considerable risk levels (High). In Africa the risk is concentrated in coastal zones and in Central Africa, from latitude 10°N to 34°S. In Europe the risk peaks in the Mediterranean and Atlantic coasts and decreases with higher latitudes; Italy, France, Spain, Portugal, Greece and Turkey have medium to high-risk levels (Fig. 2).

**Fig. 3.**
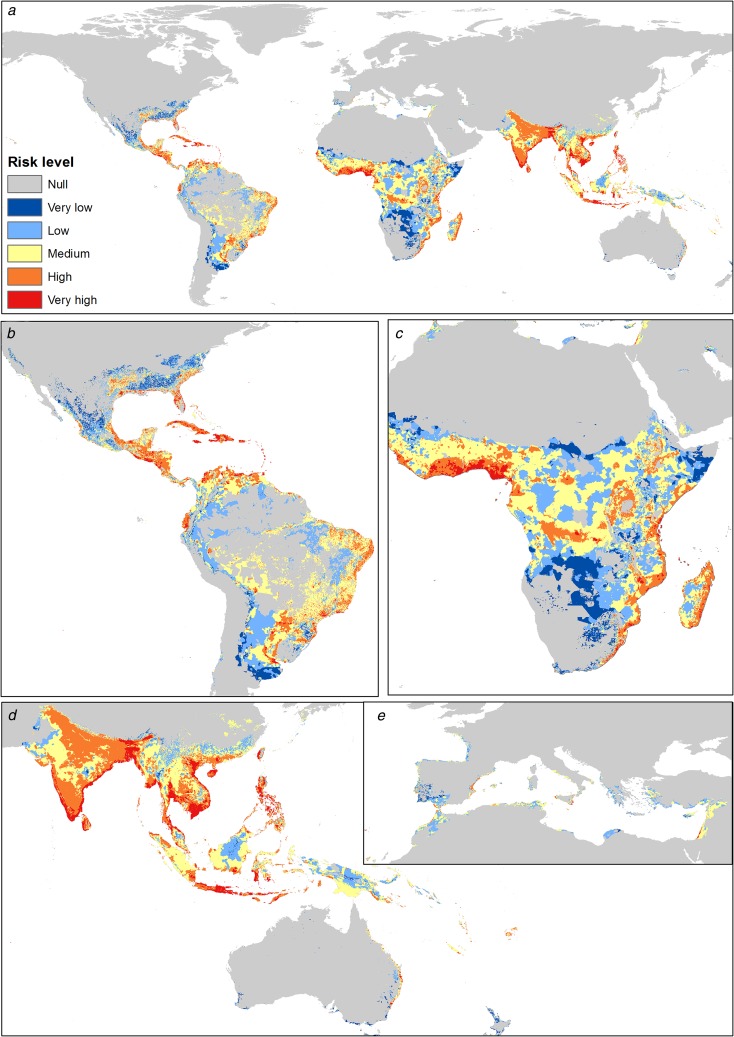
Transmission risk model of ZIKV due to the vector *Aedes aegypti*. (a) Map of the transmission risk of ZIKV worldwide by *A. aegypti*. (b) Zoom to the transmission risk map of America. (c) Zoom to the transmission risk map of Africa. (d) Zoom to the areas with higher transmission risk in Oceania. (e) Zoom to the transmission risk map of Europe.

The highest risk levels for *A. aegypti* are present mainly in tropical zones. In Asia, there are higher levels, mainly in the Indian ocean coastal zone. The most potentially affected zones in the Americas are Central America, Brazil, Colombia, Venezuela and the southern United States. In Africa, the risk is higher in both coastal zones, with some areas of high risk in the center of the continent. In Europe, the potential risk is present in Spain, France and Italy, while in Oceania there are lower risk levels (Fig. 3).

### Spatially explicit comparison of the risk zones of each vector worldwide

**Fig. 4.**
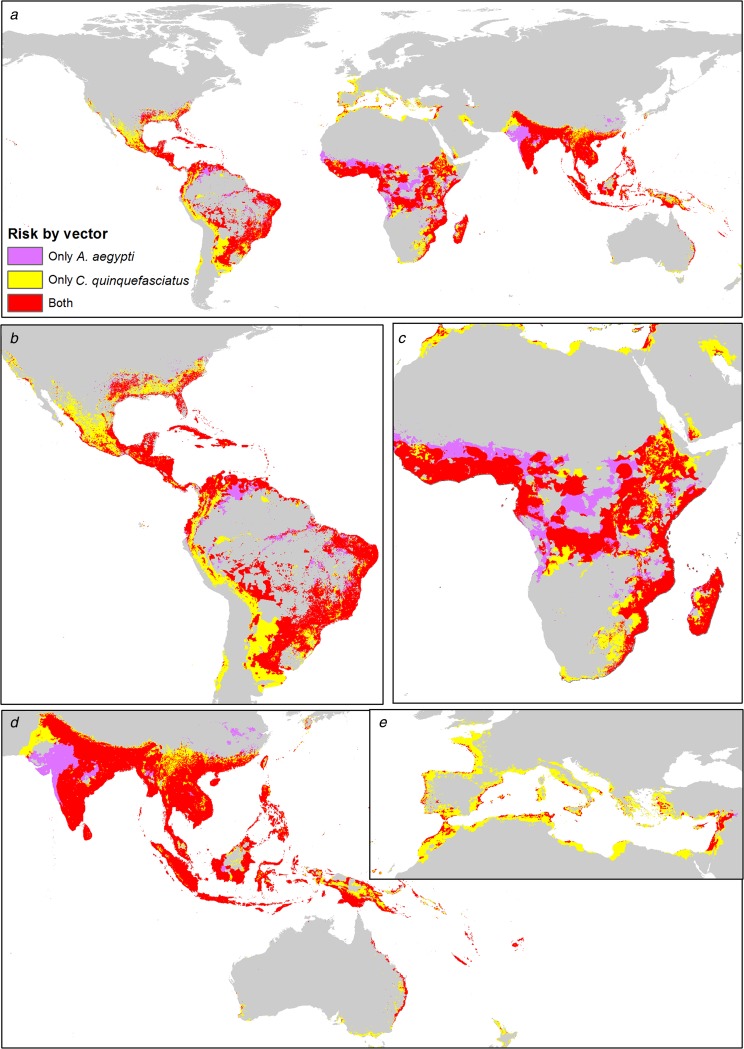
Combined risk map of *A. aegypti* and *C. quinquefasciatus* worldwide. (a) Map of the potential transmission risk of ZIKV worldwide. (b) Zoom to America. (c) Zoom to Africa. (d) Zoom to Oceania. (e) Zoom to Europe.

Both mosquitoes are present mainly in tropical zones of the world, from latitude 32°N to 32°S approximately. The influence of *C. quinquefasciatus* is preponderant at higher latitudes, from 32° to 42° in both hemispheres. In America, there is a major area associated with *C. quinquefasciatus*, which increases the ZIKV risk area. In Africa, there is a preponderance of *A. aegypti* in the risk area, with some zones in the centre of the continent without *C. quinquefasciatus*. Both mosquitoes have similar ZIKV risk areas in Asia and Oceania. In Europe, *C. quinquefasciatus* highly increases the potential ZIKV risk area (Fig. 4).

### Quantification and comparison of the people at risk associated with each vector

We quantified the population affected by each vector independently (the people in the interaction zones are attributed to both vectors). The human population exposed to high and very high ZIKV transmission risk levels due to *C. quinquefasciatus* reaches 3.66 billion people, which represents 49.7% of the world population. Approximately 4.2 billion people may be potentially exposed to ZIKV due to *C. quinquefasciatus* (Table 1; Supplementary Table S1). The ZIKV risk due to *A. aegypti* reaches 2.88 billion people under high and very high-risk levels, representing 39.4% of the world population (see Supplementary data, Table S2). The population potentially exposed to the primary vector is around 4.1 billion people.

**Table 1.** Population exposed to both mosquitoes: Quantification of the exposed human population in millions by continent and grouped by risk level of ZIKV exposure

	Very high	High	Medium	Low	Very low	Total
*Primary vector: Aedes aegypti*						
Africa	155.60	324.48	287.83	68.42	36.44	872.76
America	256.04	160.58	173.27	38.30	5.09	633.29
Asia	616.71	1348.22	586.76	42.25	0.50	2594.44
Oceania	8.72	4.15	8.19	2.59	0.32	23.96
Europa	0.86	8.51	26.97	4.32	0.39	41.04
total	1037.93	1845.93	1083.01	155.88	42.74	4165.49
*Potential vector: Culex quinquefasciatus*						
Africa	423.48	223.91	140.20	40.72	11.37	839.69
America	494.79	137.51	65.05	22.58	4.69	724.62
Asia	1706.27	570.41	134.58	48.62	4.75	2464.63
Oceania	20.97	6.61	3.25	0.69	0.26	31.79
Europe	44.68	38.42	27.94	43.70	5.62	160.36
Total	2690.19	976.87	371.03	156.31	26.69	4221.09

**Fig. 5.**
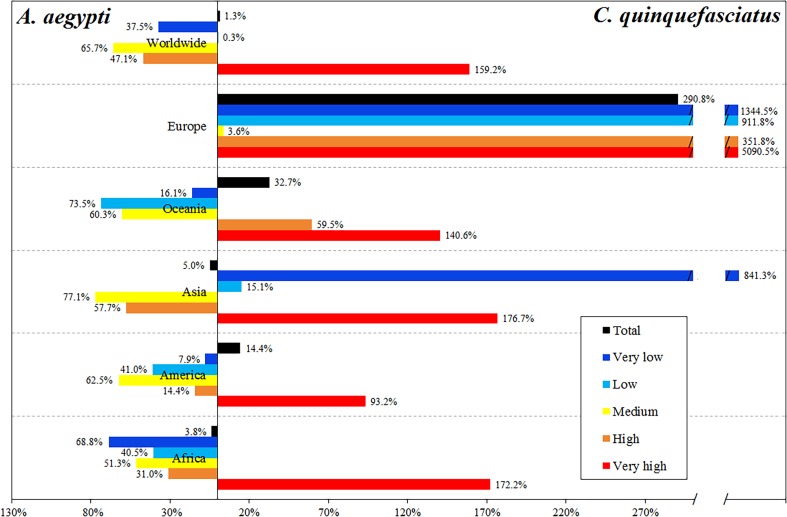
Differential in the percentage of people exposed to ZIKV by the two vectors, showing which of the vectors accounts for the difference, by continent and by risk level.

*C. quinquefasciatus* increases the total population at risk by 0.75% in relation to the risk due to *A. aegypti.* However, people at very high-risk level increase 1.59 times due to *C. quinquefasciatus*, while the people at high and medium risk levels worldwide is mainly due to *A. aegypti* (Table 1, Fig. 5).

The most affected continent is Asia, with 2.46 billion people exposed to *C. quinquefasciatus* and 2.59 billion exposed to *A. aegypti*, most of whom reside in China and India. In Africa, 839.7 and 872.76 million people are exposed to *C. quinquefasciatus* and *A. aegypti*, respectively. In the Americas, this secondary vector increases the exposed people by 14.4% (Table 1).

Europe has 160.3 million people potentially exposed to ZIKV due to *A. aegypti*; this continent has the largest increase in the population exposed considering the potential effect of *C. quinquefasciatus* (2.9 times more), concentrated in France, Spain, Italy and even the UK. Oceania, with 31.8 million people at risk due to *A. aegypti*, has 32.7% increase in the population exposed due to *C. quinquefasciatus*. In Africa and Asia, there are 2.97% more people at risk due to *A. aegypti* than to *C. quinquefasciatus* (Fig. 5).

**Fig. 6.**
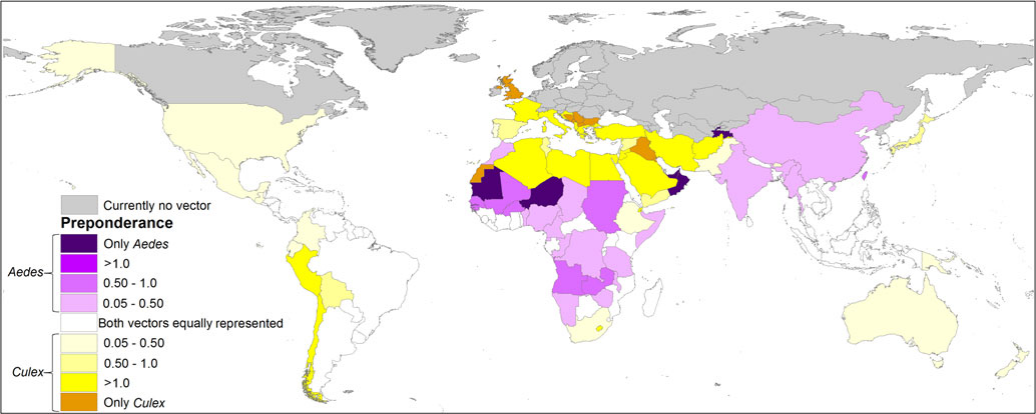
Spatially explicit differential in the percentage of people exposed to ZIKV by both vectors, showing which of the vectors accounts for the difference, by country. In grey, if the studied vectors are not present according to the models; in white, if there is no difference in the percentage of population exposed between both vectors; in purple, if *Aedes aegypti* accounts for the difference; in yellow if *Culex quinquefasciatus* accounts for the difference. The darker colour of each palette indicates that only that vector species is present in the country according to the model.

The people at very high-risk levels generally increase considerably when the risk of exposure to infected *C. quinquefasciatus* is included. In 67 countries *A. aegypti* is preponderant in the risk of ZIKV, which are mainly located in the equatorial areas, while in 83 countries the people are potentially exposed to ZIKV mainly due to *C. quinquefasciatus* (Fig. 6, Supplementary data, Tables S2 and S3).

## Discussion

### About the model

Previous studies have tested the usefulness of SDM to estimate the distribution of vectors worldwide [10, 32, 44]. A recent study suggested a protocol to combine these models with human population density, aiming to estimate the risk of transmission of vectorial infectious diseases [10]. We use this approach to evaluate the ZIKV risk due to *C. quinquefasciatus*. The present study updates the model of Alaniz *et al*. [10], because we integrate the most recent human density and count grids, sharpening the prediction for *A. aegypti* risk of ZIKV. Furthermore, we incorporate the potential vector, complementing and expanding the prediction of that previous model [10]. The distribution of *C. quinquefasciatus* was estimated by Samy *et al*. [32] using SDMs and that prediction reported the presence of the vector in some areas of Africa, Middle East Asia and India that do not coincide with our results. It is possible that the differences in the suitability map for *C. quinquefasciatus* obtained here are related to the different occurrences used by both studies. We present here the most complete *C. quinquefasciatus* occurrence database worldwide reported to this date, representing a contribution to develop future studies of this vector [30–32, 45].

We advise that our estimations correspond to ‘exposure risk’, which is related to the presence of a potentially infected vector in populated zones. However, we cannot predict the effective infection, because this could depend on several complex factors. Our modelling scenario considered only three main factors: distance from interaction zones, suitability for *C. quinquefasciatus* and human population density. However, vulnerability – a human-dependent factor and threat – a mosquito-dependent factor – could modulate these risk predictions and determine the effective infection [46, 47]. The vulnerability is influenced by poverty, sanitation, public health resources and prevention actions, aspects not taken into account in our model [48, 49]. The mosquito threat could change depending on the availability of breeding sites, related to habitat modification, abundance of females, behavioural and seasonal changes and changes in the distribution range due to climate change [30, 50, 51]. Additionally, some studies raised that infection is difficult to predict by only considering SDM of the interacting vector, being important other factor such as connectivity to areas of current virus circulation [52, 53]. However, at large scales the patterns of distribution and abundance of species are mainly explained by bioclimatic factors [54], hence our model constitutes an estimate at a global scale (coarse-grain), but the risk could be modulated at the local scale depending on the management of risk components [47]. The ranges of *C. quinquefasciatus* and *A. aegypti* are expected to increase into higher latitudes in the future due to climate change [32, 55].

The dispersal capacity of *C. quinquefasciatus* considered here was selected using reported maximum dispersal distances, which are highly variable [38–41]. In order to account for a possible seasonality in dispersal, we attributed this theoretical distance to the accumulated dispersal during half a year [56]. However, it is possible that this dispersal restriction does not exist in tropical regions where seasonality is not strong and so *C. quinquefasciatus* could disperse during the whole year, achieving longer dispersal distances, hence the risk would increase in areas located further from the interaction zones if the environmental conditions are suitable.

Our model has two main assumptions: (A) we assume that all the *A. aegypti* individuals are presumably infected across their entire distribution range, hence all the *C. quinquefasciatus* individuals which overlap with *A. aegypti* range has the same probability to become infected. We are not capable to estimate the real distribution or density of the infected individuals and additionally, the virus distribution could respond to other environmental factors which are difficult to evaluate [57]. (B) A homogeneous distribution of the available host infected with ZIKV across space, where the main source of infection of *C. quinquefasciatus* is by feeding on host an infected. There is highly difficult to estimate or interpolate the amount and the specific distribution of the host, because these values are very stochastic and dynamic through time and space [26].

### *Culex quinquefasciatus* and ZIKV: potential repercussion

The possible competence of *C. quinquefasciatus* as a secondary vector of ZIKV is a topic under study with contradictory and controversial results [20]. However, the evidence indicates that the possibility of transmission associated with this new vector may constitute a threat to public health [12–19]. The estimated distribution of *A. aegypti* spans between latitude 35° N to 35° S approximately [10, 41]. We found that the distribution of *C. quinquefasciatus* reaches latitude 42° in both hemispheres, which could expand the potential zone of influence of ZIKV to unsuitable territories for *A. aegypti*. In all continents, *C. quinquefasciatus* expands the area of ZIKV influence. The prediction of our model shows that the worldwide suitability for *C. quinquefasciatus* is higher than for *A. aegypti*, which may be related to the resistance capability to variable climatic conditions of the former, which is much more common than *A. aegypti* [24, 25]. In some countries there is presence and interaction of both vectors, while in others where *A. aegypti* is uncommon, *Culex* could expand the influence of viruses, increasing the number of countries that will have to deal with this sanitary issue in the case of an outbreak. In 2015, 49 countries reported active transmission of ZIKV [44, 58]. Previous studies indicated that around 130–170 countries/territories were at risk associated with *A. aegypti* [10, 11]. Here we found that the risk due to the primary vector coupled with this potential secondary vector, *C. quinquefasciatus*, spans 182 countries/territories.

Given that people move longer distances than mosquitoes, it is possible that infected persons could start outbreaks of endemic transmission in areas where the primary vector is absent [51] but secondary vectors such as *C. quinquefasciatus* are present, so it is imperative to reinforce educational campaigns, especially for people from non-endemic countries travelling to endemic areas, to prevent infection in those travellers [59].

We provide a risk map at 5 km^2^ pixel resolution for *A. aegypti* and *C. quinquefasciatus* as a tool for management of arboviruses and public health. The predictions presented here represent a baseline for other viruses which have both mosquitoes as vectors such as West Nile Virus and Saint Louis Encephalitis [60, 61]. We also share the complete occurrence database and SDM of *C. quinquefasciatus* to promote the development of new modelling studies, which could assess the issue of a risk considering climate change scenarios. We commend all the efforts of recent and highly relevant studies on vector competence of mosquito species associated with ZIKV, aiming to detect and clarify the competence of *C. quinquefasciatus* and other potential secondary vectors to help prevent future epidemic outbreaks; we strongly encourage these studies to continue in the future. It is necessary to ensure the control of the main vector *A. aegypti*, focusing on countries with higher poverty rates and lower sanitation conditions, avoiding potential epidemic outbreaks associated with the exposure to ZIKV vectors.

## Supplementary material

For supplementary material accompanying this paper visit https://doi.org/10.1017/S0950268818003102. click here to view supplementary material
