# Supplementary material for: Global spatial assessment of Aedes aegypti and Culex quinquefasciatus: a scenario of Zika virus exposure
Source: Epidemiol Infect. 2018 Nov 26;147:e52. doi: 10.1017/S0950268818003102 (PMC6518585; doi:10.1017/S0950268818003102)

**Supplementary data**

**Supplementary data, File 1 (description):** Table of compiled occurrences of *Culex quinquefasciatus* worldwide

| **Parameter** | **Description** |
| --- | --- |
| Archive name | C_quinque_occurrence |
| Extension | .xlsx |
| Weight | 103 kb |
| Coordinate system | Coordinate global system (decimal degrees) |
| Number of occurrences | 3866 |

**Supplementary data, Files 2, 3, 4 and 5 (description)**: 2) Distribution model of *Culex quinquefasciatus* (Probabilities multiplied by 1000) 3) Worldwide risk map associated to *C. quinquefasciatus* as downloadable raster format at 5km^2^ pixel. The risk values are classified from 0 to 5 (0=null; 1= very low; 2= low; 3= medium; 4= high; 5= very high) 4) Worldwide risk map associated to *Aedes* *aegypti* as downloadable raster format at 5km^2^ pixel. D) World risk map of both vectors considering only medium, high and very high risk levels, classified in three categories (1= Only *A. aegypti*; 2= Only *C. quinquefasciatus*; 3= Both vectors)

Supplementary data 3:

| File name | Risk_Map_Culexq.tif |
| --- | --- |
| File extension | GeoTIFF |
| Compression type | LZW |
| Coordinate system | CGS WGS 1984 |
| Datum | D WGS 1984 |
| Weight | 0.84 MB |
| Bit depth | 8 bit unsigned |
| Resolution | 5x5 km^2^ |

Supplementary data 2:

| File name | SDM_Culexq.tif |
| --- | --- |
| File extension | GeoTIFF |
| Compression type | LZW |
| Coordinate system | CGS WGS 1984 |
| Datum | D WGS 1984 |
| Weight | 39.2 MB |
| Bit depth | 8 bit unsigned |
| Resolution | 1x1 km^2^ |

Supplementary data 5:

| File name | Risk by Vector.tif |
| --- | --- |
| File extension | GeoTIFF |
| Compression type | LZW |
| Coordinate system | CGS WGS 1984 |
| Datum | D WGS 1984 |
| Weight | 0.62 MB |
| Bit depth | 32 bit unsigned |
| Resolution | 1x1 km^2^ |

Supplementary data 4:

| File name | Risk_Map_Aedesa_2015.tif |
| --- | --- |
| File extension | GeoTIFF |
| Compression type | LZW |
| Coordinate system | CGS WGS 1984 |
| Datum | D WGS 1984 |
| Weight | 0.75 MB |
| Bit depth | 8 bit unsigned |
| Resolution | 5x5 km^2^ |

**Supplementary data, Figure 1:** Correlogram panel of *C. quinquefasciatus* suitability model variables. The panel shows only the selected variables in the final model. The upper right boxes show the absolute correlation index (r) and its p-value. The diagonal boxes contain the result of Shapiro-Wilk test and the histograms of each variable. The lower left boxes show the correlation between pairs of variables’ scatter plots.


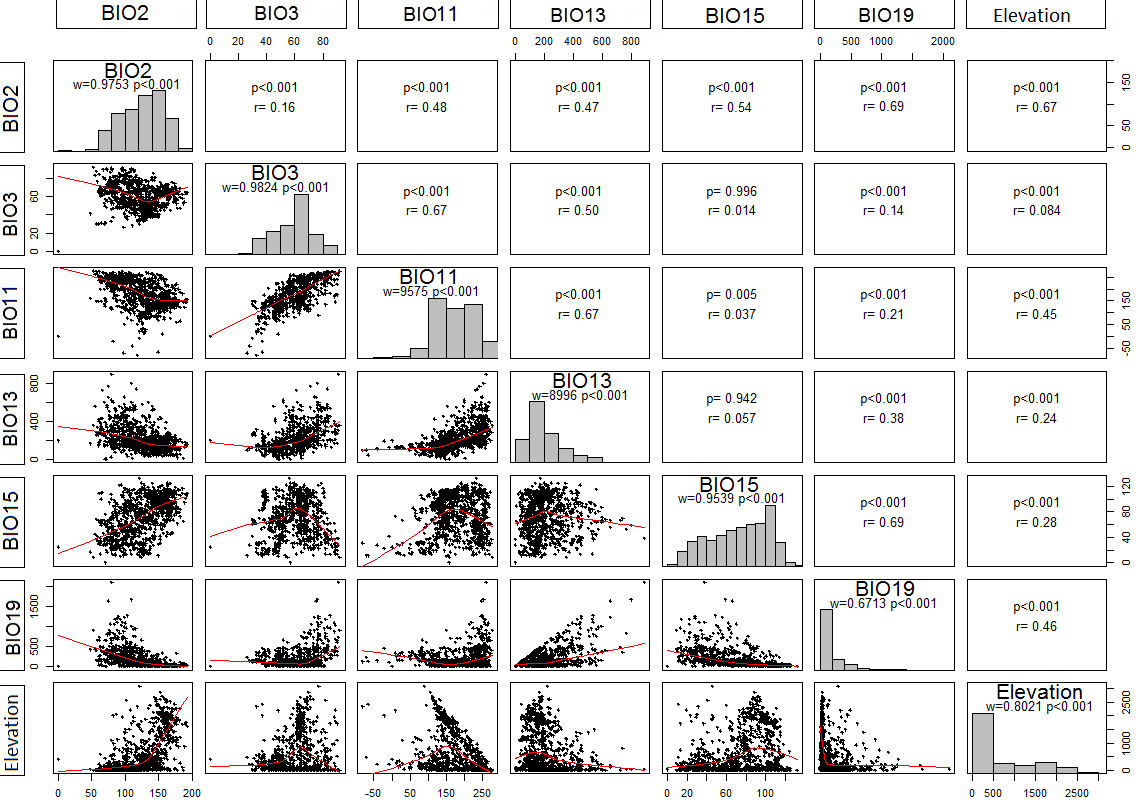


**Supplementary data, Figure 2:** Response curves and variables’ contribution in the model of *Culex quinquefasciatus*: the response curves shows how the logistic prediction of suitability of *C. quinquefasciatus* chance as each environmental variable is varied. Left panels: Response curves in relation to the corresponding variable when all the other environmental variables are maintained invariant. Right: Response curves using only the corresponding variable. PC: Percentage of contribution; PI: Permutation importance.


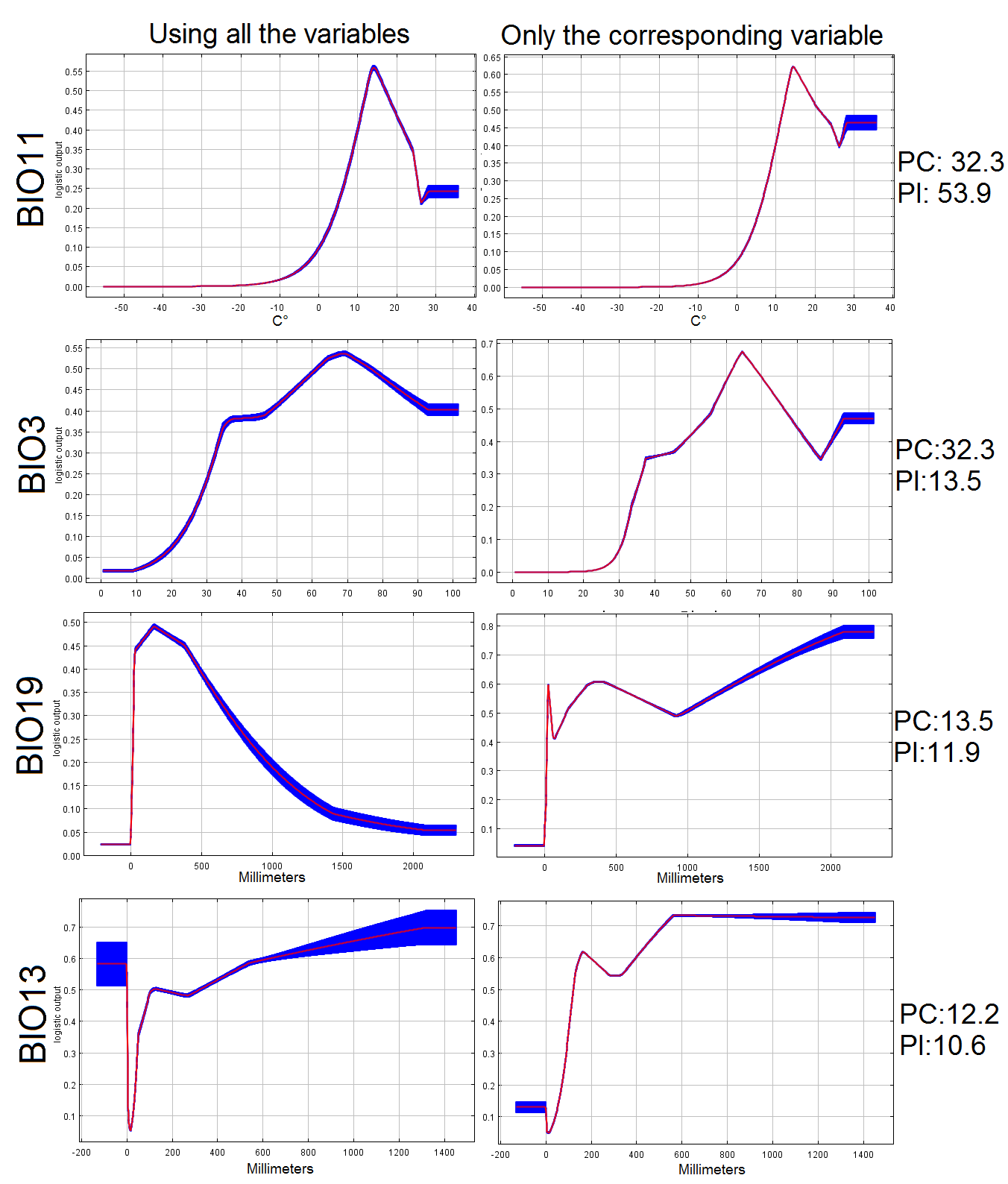


**Supplementary data, Figure 3:** Curves of omission and ROC curve. Left: Curve of omission rate, which test the omission rate and the predicted area as a function of the cumulative threshold, averaged over the replicate number (50). Right: Curve of the receiver operating characteristic, which compares sensitivity and specificity of the model to assess the accuracy of the predicted area of the model.


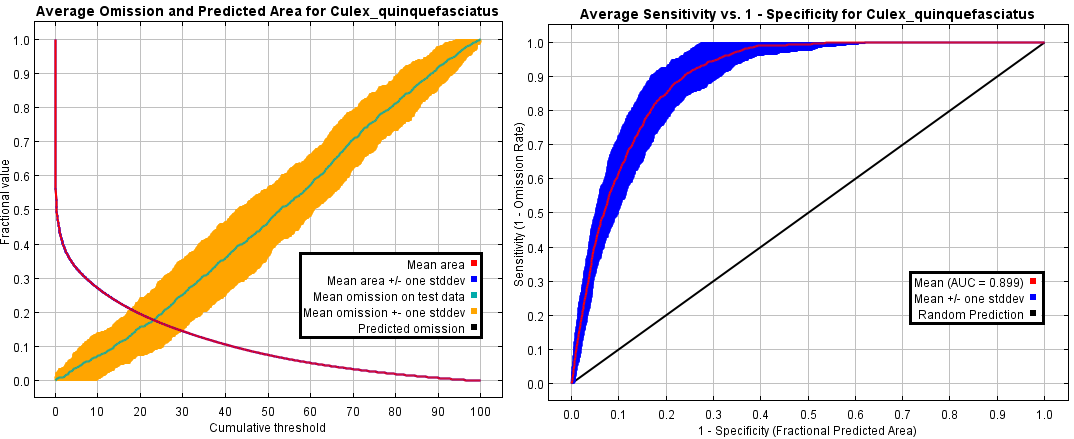


**Supplementary data, Figure 4:** Uncertainty expressed in percentage calculated from the standard deviation of the predicted suability of *C. quinquefasciatus*.


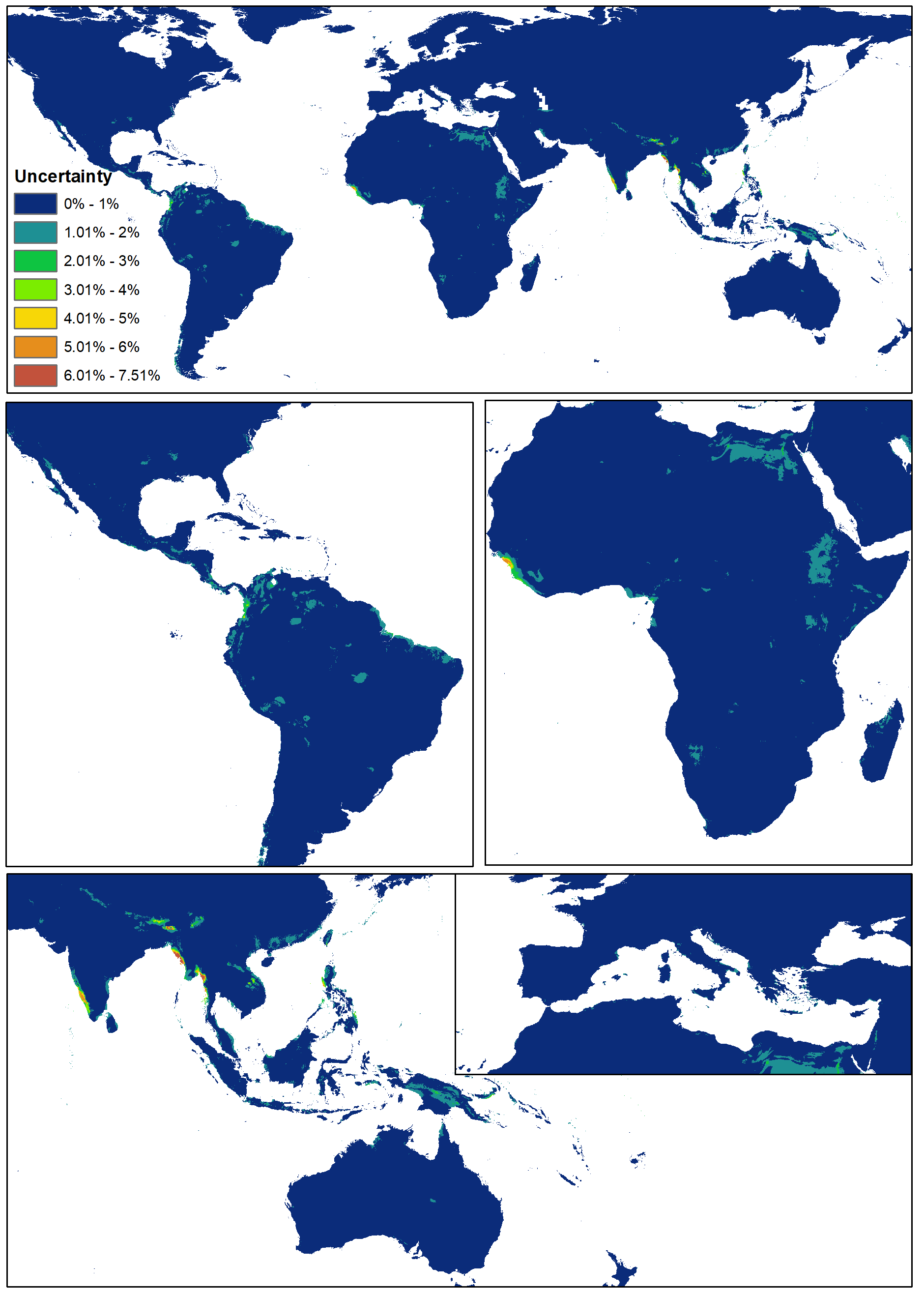


**Supplementary data, Figure 5:** Uncertainty expressed in percentage calculated from the standard deviation of the predicted suability of *C. quinquefasciatus*.


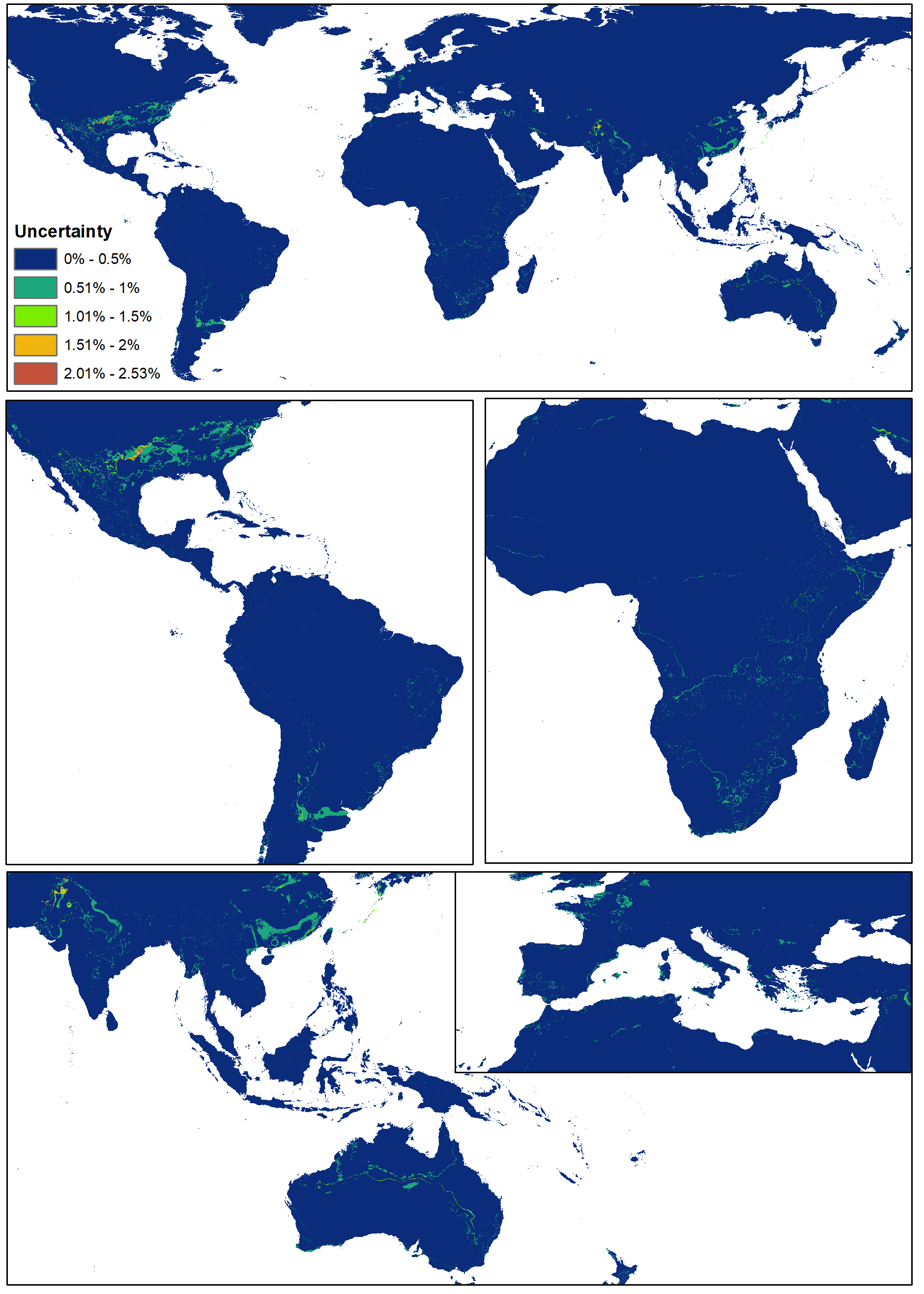


**Supplementary data, Figure 6:** Test of importance of the variables. A) Jackknife analysis developed in MaxEnt model, to evaluate the contribution of the variables to the model, testing the variable by itself (as the only variable) and the reduction of AUC when the variable is not included B) Percentage of contribution and permutation importance of the variable in the model. C) Partial Least Square regression of the model variables: *a posteriori* test which tests the prediction of MaxEnt using the environmental variables as predictors and the suitability (Y) as the response variable.


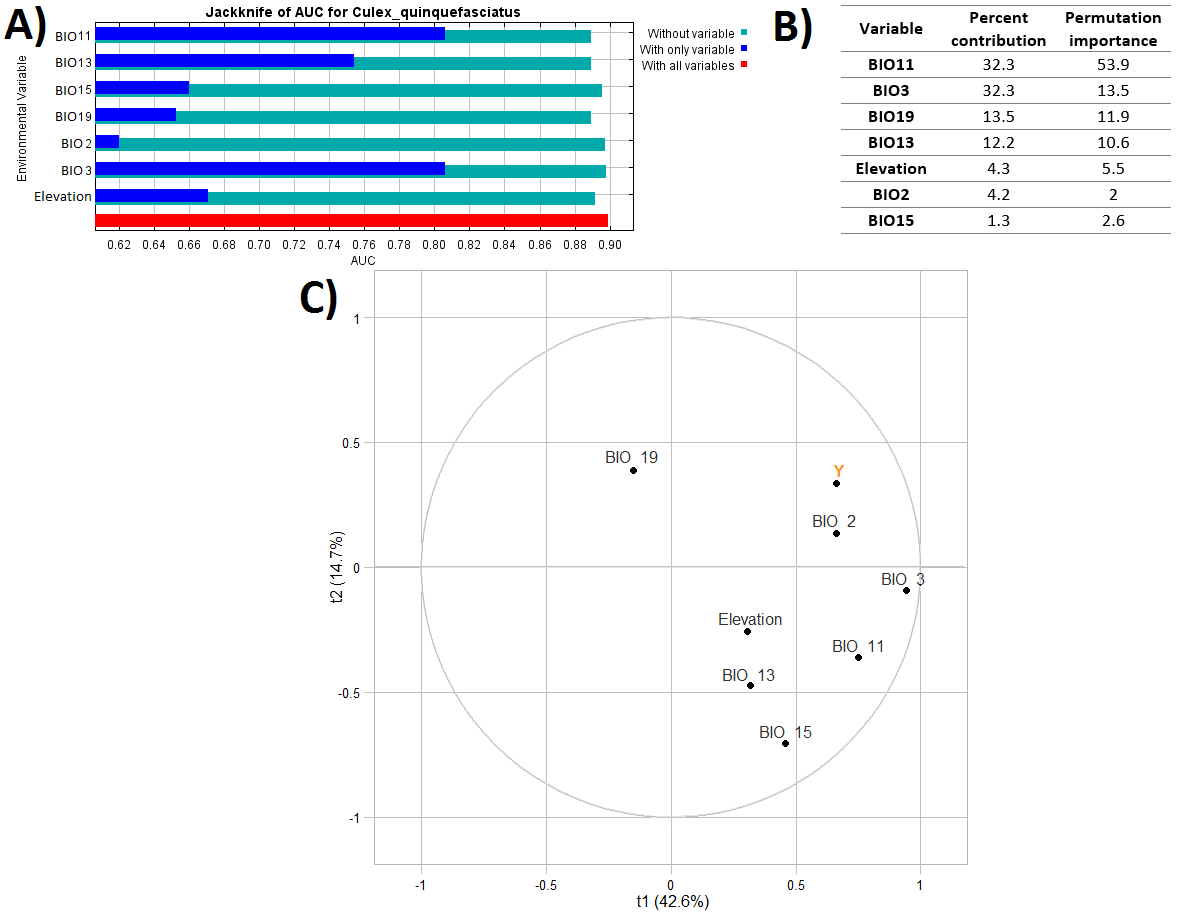


**Supplementary data, Figure 7:** Parameters used to estimate the risk of ZIKV transmission associated to *Culex quinquefasciatus*. A) Distance map to the interaction zones (DIZ) between *C. quinquefasciatus* and *Aedes aegypti.* B) Reclassified suitability model of *C. quinquefasciatus.* C) Human population density grid of NASA (2016)


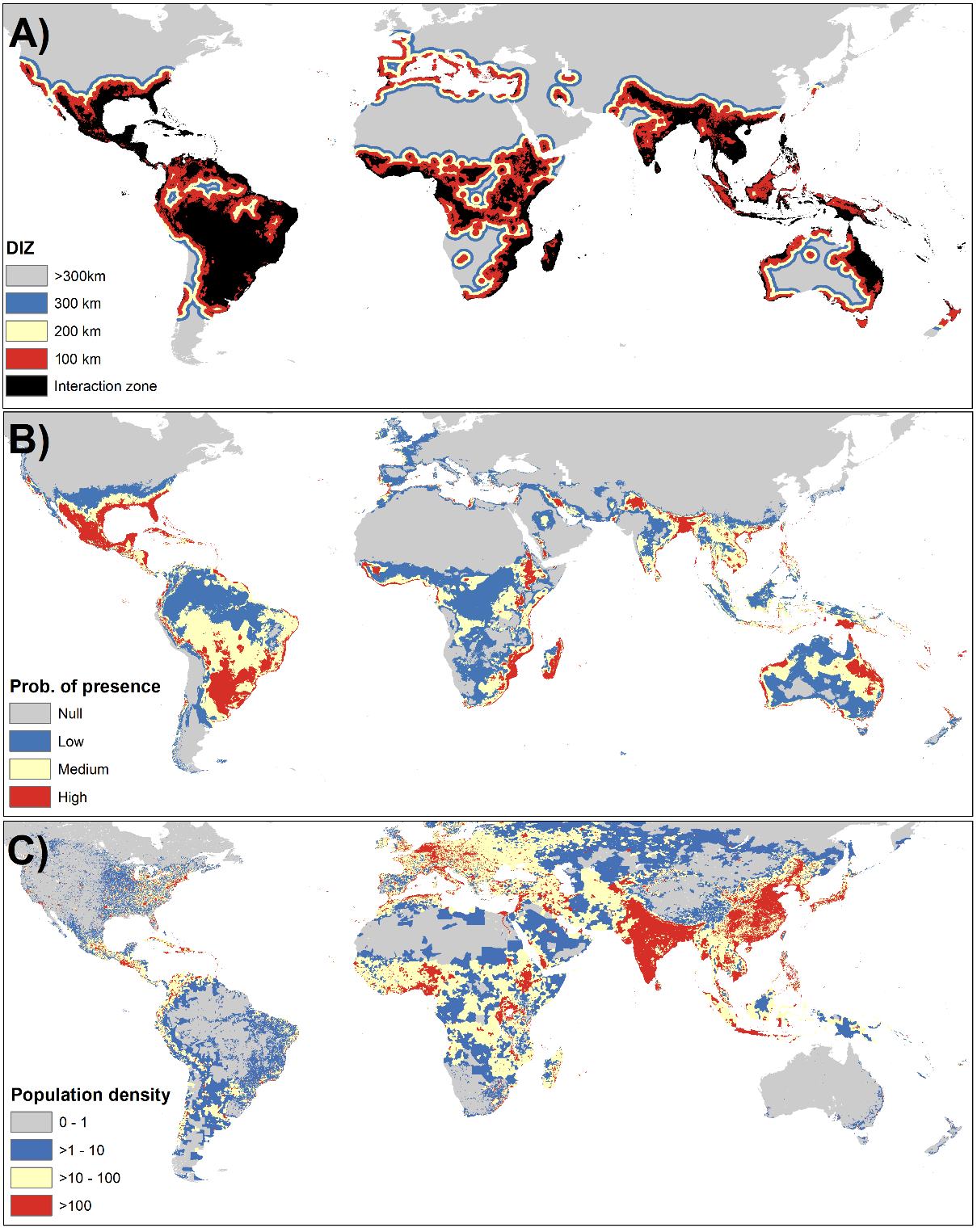


**Supplementary data, Figure 8**: Schemes of the raster multiplication process and the probability of spatial interaction between vectors. Left: double entry matrix which links both probabilities of presence’s grids of each vector. The values (0 to 3) correspond to the reclassified levels of the suitability variable. Right: Probability of vector zonal interaction according to the result of the grid multiplication.


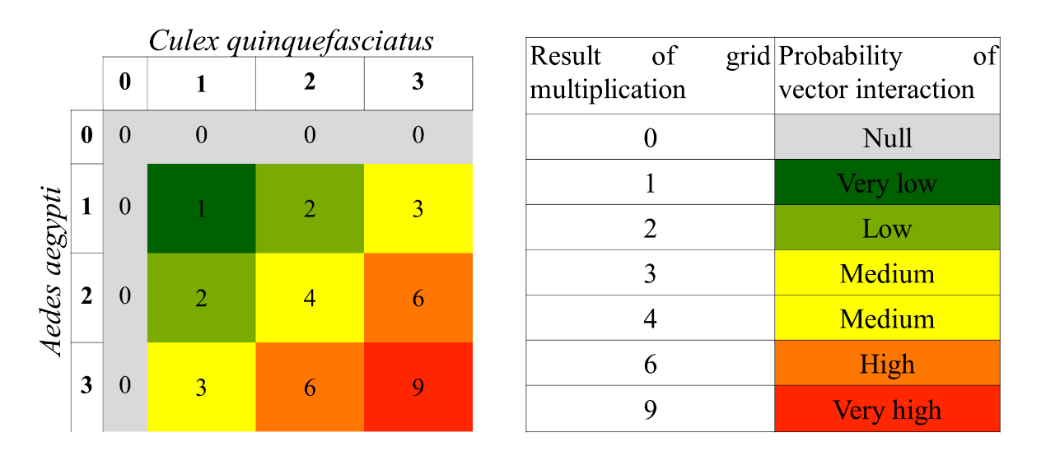


**Supplementary data, Figure 9**: Schemes of the raster multiplication process and the risk of ZIKV transmission associated to *Culex quinquefasciatus*. Step one: double entry matrix of the grid multiplication of: the distance from zones of interaction [DIZ] and the probability of presence (suitability or potential abundance) of *Culex quinquefasciatus.* Step two: double entry matrix of the grid multiplication process of: the population density and the grid resulting from step one. Step 3: reclassification of the transmission risk into five risk levels, from 0 to 5 (null to very high).


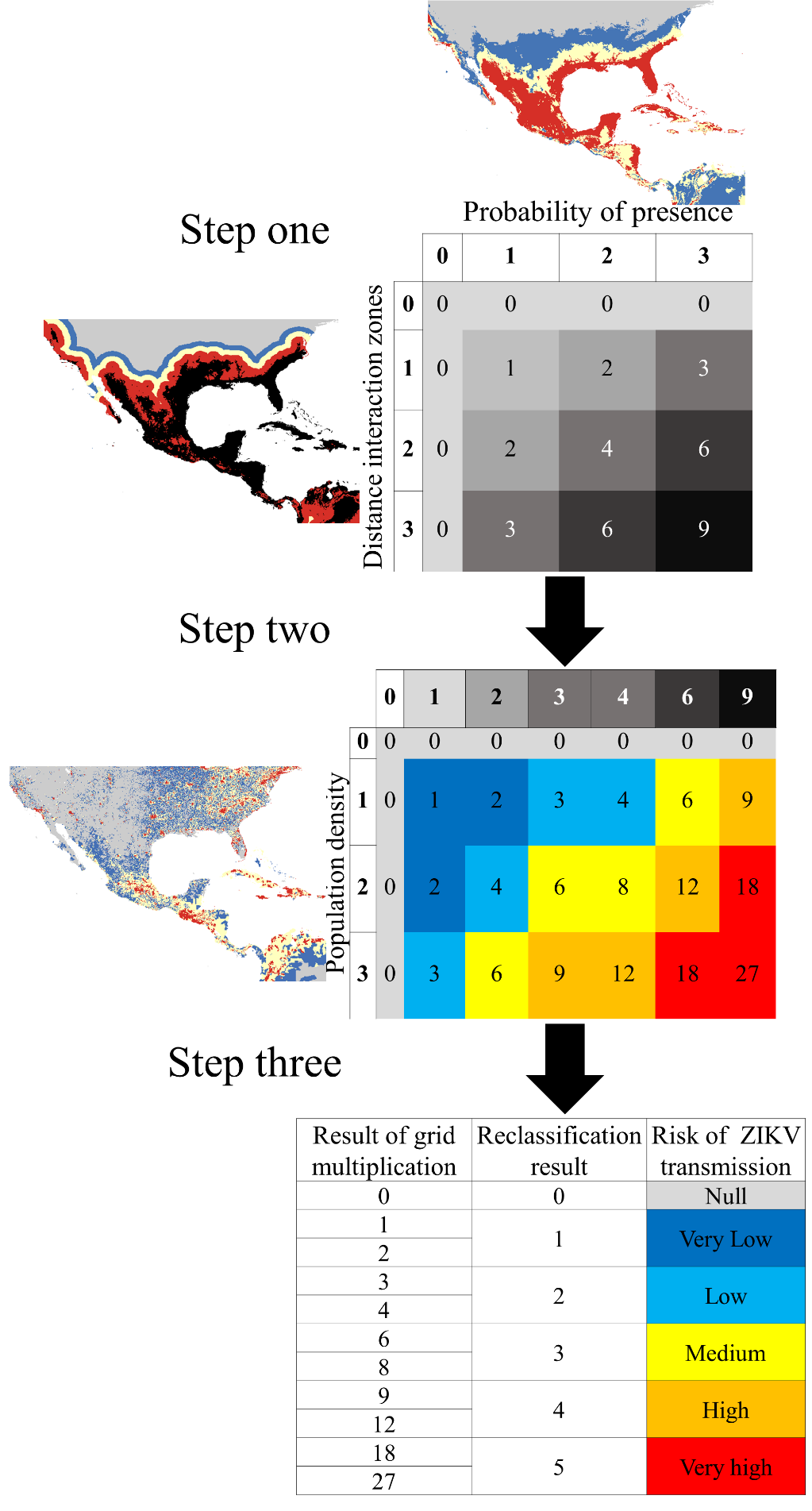

Supplement: Supplementary file 1 [file S0950268818003102sup001.zip › S0950268818003102sup001/Supplementary_data_Fig_1_to_9_R2.docx]
