# Supplementary material for: Global spatial assessment of Aedes aegypti and Culex quinquefasciatus: a scenario of Zika virus exposure
Source: Epidemiol Infect. 2018 Nov 26;147:e52. doi: 10.1017/S0950268818003102 (PMC6518585; doi:10.1017/S0950268818003102)
Supplement: Supplementary file 1 [file S0950268818003102sup001.zip › S0950268818003102sup001/Supplementary_data_table_4.docx]

**Supplementary table 4:** Population potentially exposed to ZIKV based on the model of exposure to *Aedes aegypti*, classified by risk level, country and continent. The column “% *Aedes aegypti*” represents the percentage of the population potentially affected by country due to the contact with vector. Countries not listed do present null risk of ZIKV due to this vector, according to our model.

| **Region** | **Sub Region** | **Country** | **Very high** | **High** | **Medium** | **Low** | **Very low** | **Total** | **Population 2015** | ***% Aedes aegypti*** |
| --- | --- | --- | --- | --- | --- | --- | --- | --- | --- | --- |
| **Africa** | Central Africa | Sao Tome and Principe | 145613 | 34506 | 1276 | 0 | 0 | 181395 | 202781 | 89.45% |
|  |  | Gabon | 701561 | 806103 | 155387 | 53233 | 0 | 1716284 | 1725292 | 99.48% |
|  |  | Equatorial Guinea | 440615 | 324220 | 14706 | 4025 | 0 | 783566 | 799372 | 98.02% |
|  |  | Congo | 172555 | 1353395 | 678455 | 1313676 | 6541 | 3524622 | 4671142 | 75.46% |
|  |  | Central African Republic | 72739 | 1427712 | 2019916 | 886557 | 10765 | 4417689 | 4803082 | 91.98% |
|  |  | Chad | 0 | 119862 | 4487712 | 1754196 | 272674 | 6634444 | 13605625 | 48.76% |
|  |  | Cameroon | 3528350 | 9368012 | 8866383 | 680413 | 3057 | 22446215 | 23393129 | 95.95% |
|  |  | Angola | 294398 | 2382285 | 13942806 | 5310743 | 822556 | 22752788 | 22819926 | 99.71% |
|  |  | Democratic Republic of the Congo | 2242380 | 32833901 | 34135383 | 5770086 | 5086 | 74986836 | 77266814 | 97.05% |
|  | East Africa | Djibouti | 0 | 0 | 0 | 5971 | 64 | 6035 | 899658 | 0.67% |
|  |  | Seychelles | 75888 | 0 | 0 | 0 | 0 | 75888 | 93754 | 80.94% |
|  |  | Mayotte | 188728 | 0 | 0 | 0 | 0 | 188728 | 233993 | 80.66% |
|  |  | Comoros | 603895 | 58121 | 16606 | 0 | 0 | 678622 | 770058 | 88.13% |
|  |  | Reunion | 573810 | 84387 | 82427 | 2263 | 0 | 742887 | 895099 | 82.99% |
|  |  | Mauritius | 1139608 | 74412 | 9226 | 0 | 0 | 1223246 | 1253581 | 97.58% |
|  |  | Eritrea | 0 | 147066 | 1360200 | 1234615 | 744 | 2742625 | 6737634 | 40.71% |
|  |  | Somalia | 1095351 | 2284043 | 1768326 | 2065262 | 968454 | 8181436 | 11122711 | 73.56% |
|  |  | Burundi | 581322 | 8301382 | 898037 | 593 | 1596 | 9782930 | 10812619 | 90.48% |
|  |  | Rwanda | 0 | 6068919 | 4953057 | 6557 | 229 | 11028762 | 12428005 | 88.74% |
|  |  | Zambia | 0 | 53378 | 4905213 | 5260915 | 2259729 | 12479235 | 15519604 | 80.41% |
|  |  | Zimbabwe | 6988 | 412195 | 6819246 | 5910864 | 192014 | 13341307 | 15046102 | 88.67% |
|  |  | Malawi | 2421786 | 7908372 | 4866904 | 541187 | 29212 | 15767461 | 17308685 | 91.10% |
|  |  | Madagascar | 3336623 | 12198529 | 6793982 | 1071687 | 251 | 23401072 | 24235390 | 96.56% |
|  |  | Mozambique | 7365661 | 10196113 | 6972241 | 975363 | 10760 | 25520138 | 27121827 | 94.09% |
|  |  | Uganda | 34824 | 29961863 | 7471016 | 103215 | 59 | 37570977 | 40141262 | 93.60% |
|  |  | Kenya | 2716032 | 17991714 | 20242729 | 2127912 | 181381 | 43259768 | 46748617 | 92.54% |
|  |  | United Republic of Tanzania | 9786124 | 19162093 | 14130112 | 3441401 | 76072 | 46595802 | 52290796 | 89.11% |
|  |  | Ethiopia | 0 | 28157508 | 38787692 | 7851350 | 346030 | 75142580 | 98942102 | 75.95% |
|  | North Africa | Egypt | 0 | 0 | 1342492 | 0 | 0 | 1342492 | 84705681 | 1.58% |
|  |  | Libyan Arab Jamahiriya | 0 | 29340 | 1341785 | 609845 | 21694 | 2002664 | 6317080 | 31.70% |
|  |  | Tunisia | 0 | 10067 | 4575229 | 568117 | 0 | 5153413 | 11235248 | 45.87% |
|  |  | Algeria | 0 | 2549603 | 6455677 | 361293 | 160 | 9366733 | 40633464 | 23.05% |
|  |  | Sudan | 1666291 | 10130472 | 2094686 | 331524 | 14222973 | 28445946 | 39613217 | 71.81% |
|  |  | Morocco | 4192862 | 12871943 | 2732447 | 0 | 14197252 | 33994504 | 34377511 | 98.89% |
|  | Southern Africa | Lesotho | 0 | 0 | 119069 | 12152 | 1695 | 132916 | 2120116 | 6.27% |
|  |  | Namibia | 0 | 0 | 410941 | 137716 | 108125 | 656782 | 2392370 | 27.45% |
|  |  | Swaziland | 193896 | 685549 | 153977 | 18444 | 421 | 1052287 | 1285519 | 81.86% |
|  |  | Botswana | 0 | 0 | 495124 | 621221 | 239781 | 1356126 | 2056370 | 65.95% |
|  |  | South Africa | 7163945 | 9938195 | 20602239 | 4270167 | 1941849 | 43916395 | 53491333 | 82.10% |
|  | West Africa | Saint Helena | 0 | 0 | 2450 | 314 | 0 | 2764 | 4124 | 67.02% |
|  |  | Niger | 0 | 0 | 80607 | 163547 | 590 | 244744 | 19899120 | 1.23% |
|  |  | Cape Verde | 135073 | 189952 | 78922 | 859 | 0 | 404806 | 508315 | 79.64% |
|  |  | Mauritania | 0 | 0 | 222989 | 719521 | 182283 | 1124793 | 4067564 | 27.65% |
|  |  | Guinea-Bissau | 535032 | 409870 | 670921 | 2537 | 0 | 1618360 | 1787793 | 90.52% |
|  |  | Gambia | 336101 | 993682 | 369914 | 201921 | 0 | 1901618 | 1970081 | 96.52% |
|  |  | Liberia | 1504294 | 653984 | 1680656 | 163752 | 0 | 4002686 | 4503439 | 88.88% |
|  |  | Sierra Leone | 2490985 | 2359803 | 1218934 | 31919 | 0 | 6101641 | 6318575 | 96.57% |
|  |  | Togo | 3382587 | 1716993 | 2063487 | 0 | 0 | 7163067 | 7170797 | 99.89% |
|  |  | Benin | 5853763 | 1167135 | 3534308 | 120981 | 39680 | 10715867 | 10879828 | 98.49% |
|  |  | Guinea | 1993726 | 2417104 | 5436707 | 1001103 | 26146 | 10874786 | 12347766 | 88.07% |
|  |  | Senegal | 4018615 | 3787704 | 3575478 | 1237767 | 39807 | 12659371 | 14967446 | 84.58% |
|  |  | Mali | 0 | 3690541 | 3804003 | 5335544 | 211061 | 13041149 | 16258587 | 80.21% |
|  |  | Burkina Faso | 0 | 6472958 | 8256388 | 1624520 | 19870 | 16373736 | 17914625 | 91.40% |
|  |  | Cote d'Ivoire | 8959441 | 8095012 | 5113339 | 104760 | 0 | 22272552 | 23254184 | 95.78% |
|  |  | Ghana | 18049638 | 6030648 | 3091945 | 159400 | 0 | 27331631 | 27409893 | 99.71% |
|  |  | Nigeria | 57594349 | 58568122 | 23923169 | 4251734 | 7 | 144337381 | 183523432 | 78.65% |
| **America** | Caribbean | Montserrat | 0 | 3151 | 0 | 0 | 0 | 3151 | 5176 | 60.88% |
|  |  | Saint Barthelemy | 10383 | 0 | 0 | 0 | 0 | 10383 | 10500 | 98.89% |
|  |  | Anguilla | 7686 | 0 | 0 | 0 | 0 | 7686 | 14614 | 52.59% |
|  |  | Turks and Caicos Islands | 14283 | 2149 | 0 | 0 | 0 | 16432 | 34339 | 47.85% |
|  |  | British Virgin Islands | 24963 | 334 | 0 | 0 | 0 | 25297 | 28800 | 87.84% |
|  |  | Cayman Islands | 23141 | 4253 | 0 | 0 | 0 | 27394 | 59967 | 45.68% |
|  |  | Saint Martin | 27423 | 0 | 0 | 0 | 0 | 27423 | 74853 | 36.64% |
|  |  | Saint Kitts and Nevis | 46165 | 884 | 0 | 0 | 0 | 47049 | 55376 | 84.96% |
|  |  | Dominica | 35317 | 29806 | 0 | 0 | 0 | 65123 | 72680 | 89.60% |
|  |  | United States Virgin Islands | 71242 | 2421 | 0 | 0 | 0 | 73663 | 103574 | 71.12% |
|  |  | Antigua and Barbuda | 76035 | 4504 | 1700 | 0 | 0 | 82239 | 91822 | 89.56% |
|  |  | Saint Vincent and the Grenadines | 82838 | 8001 | 0 | 0 | 0 | 90839 | 109374 | 83.05% |
|  |  | Grenada | 75808 | 18345 | 0 | 0 | 0 | 94153 | 106694 | 88.25% |
|  |  | Aruba | 89480 | 6860 | 262 | 0 | 0 | 96602 | 103889 | 92.99% |
|  |  | Saint Lucia | 158803 | 0 | 0 | 0 | 0 | 158803 | 184937 | 85.87% |
|  |  | Netherlands Antilles | 133738 | 33153 | 3488 | 0 | 0 | 170379 | 227049 | 75.04% |
|  |  | Barbados | 244198 | 0 | 0 | 0 | 0 | 244198 | 287482 | 84.94% |
|  |  | Bahamas | 186992 | 61645 | 3462 | 0 | 0 | 252099 | 387549 | 65.05% |
|  |  | Martinique | 365938 | 8286 | 0 | 0 | 0 | 374224 | 405688 | 92.24% |
|  |  | Guadeloupe | 366501 | 12901 | 0 | 0 | 0 | 379402 | 470168 | 80.69% |
|  |  | Trinidad and Tobago | 1043066 | 139083 | 35495 | 1150 | 0 | 1218794 | 1346697 | 90.50% |
|  |  | Jamaica | 2458371 | 201946 | 9091 | 0 | 0 | 2669408 | 2813276 | 94.89% |
|  |  | Puerto Rico | 3063109 | 370944 | 72348 | 0 | 0 | 3506401 | 3680058 | 95.28% |
|  |  | Dominican Republic | 8693294 | 1462870 | 156906 | 16953 | 251 | 10330274 | 10652135 | 96.98% |
|  |  | Cuba | 6711679 | 4195975 | 14022 | 0 | 0 | 10921676 | 11248783 | 97.09% |
|  |  | Haiti | 10348316 | 536297 | 34990 | 3503 | 0 | 10923106 | 10980961 | 99.47% |
|  | Central America | Bermuda | 16600 | 8104 | 0 | 0 | 0 | 24704 | 65578 | 37.67% |
|  |  | Belize | 0 | 275697 | 60793 | 4277 | 0 | 340767 | 347598 | 98.03% |
|  |  | Panama | 1866146 | 1032195 | 606752 | 94552 | 1380 | 3601025 | 3987866 | 90.30% |
|  |  | Costa Rica | 296366 | 2757163 | 1201749 | 94162 | 5704 | 4355144 | 5001657 | 87.07% |
|  |  | Nicaragua | 3039009 | 2759607 | 382319 | 993 | 0 | 6181928 | 6256510 | 98.81% |
|  |  | El Salvador | 5360037 | 855652 | 47947 | 3843 | 0 | 6267479 | 6426002 | 97.53% |
|  |  | Honduras | 3720861 | 3571558 | 1057272 | 55098 | 370 | 8405159 | 8423917 | 99.78% |
|  |  | Guatemala | 2515627 | 8727414 | 2543235 | 74225 | 0 | 13860501 | 16255094 | 85.27% |
|  | Northern America | Mexico | 11819574 | 22408390 | 49825518 | 10952411 | 1611749 | 96617642 | 125235587 | 77.15% |
|  |  | United States | 29338041 | 24966058 | 54911206 | 12782461 | 2543480 | 124541246 | 325127634 | 38.31% |
|  | South America | French Guiana | 102893 | 50566 | 36350 | 2396 | 0 | 192205 | 261729 | 73.44% |
|  |  | Suriname | 347265 | 93674 | 63083 | 30 | 0 | 504052 | 548456 | 91.90% |
|  |  | Guyana | 467827 | 98947 | 25939 | 3619 | 0 | 596332 | 807611 | 73.84% |
|  |  | Chile | 0 | 34207 | 1401516 | 371964 | 5847 | 1813534 | 17924062 | 10.12% |
|  |  | Uruguay | 1900707 | 754674 | 224566 | 113053 | 36672 | 3029672 | 3429997 | 88.33% |
|  |  | Peru | 231711 | 977770 | 2315177 | 1583501 | 102266 | 5210425 | 31161167 | 16.72% |
|  |  | Bolivia | 1725219 | 913902 | 2051594 | 448592 | 116466 | 5255773 | 11024522 | 47.67% |
|  |  | Paraguay | 4060166 | 2476901 | 471507 | 9668 | 0 | 7018242 | 7032942 | 99.79% |
|  |  | Ecuador | 4700973 | 3293118 | 1479563 | 513993 | 72010 | 10059657 | 16225691 | 62.00% |
|  |  | Venezuela | 11921065 | 15114120 | 3726108 | 482854 | 13026 | 31257173 | 31292702 | 99.89% |
|  |  | Argentina | 16948448 | 6754740 | 8004552 | 2820705 | 332045 | 34860490 | 42154914 | 82.70% |
|  |  | Colombia | 3456485 | 11949384 | 17798724 | 3387782 | 70199 | 36662574 | 49529208 | 74.02% |
|  |  | Brazil | 117850428 | 43607010 | 24706566 | 4476319 | 176144 | 190816467 | 203657210 | 93.69% |
| **ASIA** | East Asia | Korea, Republic of | 0 | 0 | 10031 | 34874 | 0 | 44905 | 50293439 | 0.09% |
|  |  | Macau | 0 | 0 | 131728 | 0 | 0 | 131728 | 587606 | 22.42% |
|  |  | Japan | 0 | 24894 | 5196908 | 179603 | 150 | 5401555 | 126818019 | 4.26% |
|  |  | Hong Kong | 0 | 1156095 | 4475739 | 0 | 0 | 5631834 | 7313557 | 77.01% |
|  |  | Taiwan | 11372692 | 5478596 | 6024382 | 58871 | 10294 | 22944835 | 23381038 | 98.13% |
|  |  | China | 11402446 | 99446910 | 157867893 | 18544806 | 145248 | 287407303 | 1401586609 | 20.51% |
|  | South Asia | Maldives | 1151 | 33 | 0 | 0 | 0 | 1184 | 357981 | 0.33% |
|  |  | Bhutan | 74413 | 137314 | 110968 | 91074 | 9715 | 423484 | 776461 | 54.54% |
|  |  | Afghanistan | 0 | 13401 | 1053999 | 87228 | 1760 | 1156388 | 32006788 | 3.61% |
|  |  | Iran (Islamic Republic of) | 0 | 248598 | 3518213 | 978790 | 23643 | 4769244 | 79476308 | 6.00% |
|  |  | Sri Lanka | 13817707 | 5523882 | 482158 | 28459 | 0 | 19852206 | 21611842 | 91.86% |
|  |  | Nepal | 350637 | 19783136 | 7464020 | 458793 | 1685 | 28058271 | 28440629 | 98.66% |
|  |  | Pakistan | 0 | 87118878 | 44350137 | 2889860 | 68026 | 134426901 | 188144040 | 71.45% |
|  |  | Bangladesh | 85957619 | 64420836 | 132848 | 0 | 0 | 150511303 | 160411249 | 93.83% |
|  |  | India | 165421502 | 871670362 | 231570956 | 7695804 | 95532 | 1276454156 | 1282390303 | 99.54% |
|  | Southeast Asia | christmas island | 0 | 1153 | 0 | 0 | 0 | 1153 | 2072 | 55.65% |
|  |  | Brunei Darussalam | 0 | 348126 | 40675 | 5127 | 0 | 393928 | 428539 | 91.92% |
|  |  | Timor-Leste | 596932 | 463933 | 75019 | 4643 | 0 | 1140527 | 1172668 | 97.26% |
|  |  | Singapore | 2292020 | 997121 | 0 | 160 | 0 | 3289301 | 5618866 | 58.54% |
|  |  | Lao People's Democratic Republic | 102479 | 2272577 | 4480364 | 135972 | 3789 | 6995181 | 7019652 | 99.65% |
|  |  | Cambodia | 11948515 | 3195496 | 392314 | 46769 | 0 | 15583094 | 15677059 | 99.40% |
|  |  | Malaysia | 7491395 | 16143702 | 5395702 | 568106 | 9454 | 29608359 | 30651176 | 96.60% |
|  |  | Burma | 12316007 | 16945088 | 16588408 | 3618802 | 33065 | 49501370 | 54751920 | 90.41% |
|  |  | Thailand | 34970548 | 20738616 | 10554069 | 221679 | 2313 | 66487225 | 67400746 | 98.64% |
|  |  | Viet Nam | 71078121 | 16024038 | 3124104 | 94782 | 198 | 90321243 | 93386630 | 96.72% |
|  |  | Philippines | 67608904 | 18520320 | 4530262 | 268134 | 4014 | 90931634 | 101802706 | 89.32% |
|  |  | Indonesia | 118287389 | 91037562 | 38156678 | 3242827 | 55272 | 250779728 | 255708785 | 98.07% |
|  | Western Asia | Oman | 0 | 0 | 0 | 438 | 0 | 438 | 4490541 | 0.01% |
|  |  | Georgia | 0 | 0 | 0 | 3751 | 0 | 3751 | 3999812 | 0.09% |
|  |  | Iraq | 0 | 0 | 3522 | 1000 | 0 | 4522 | 35766702 | 0.01% |
|  |  | United Arab Emirates | 0 | 0 | 0 | 9047 | 0 | 9047 | 9156963 | 0.10% |
|  |  | Tajikistan | 0 | 0 | 47290 | 1340 | 0 | 48630 | 8481855 | 0.57% |
|  |  | Saudi Arabia | 0 | 0 | 58056 | 60343 | 270 | 118669 | 29897741 | 0.40% |
|  |  | Cyprus | 0 | 28914 | 1000780 | 108235 | 16095 | 1154024 | 1164695 | 99.08% |
|  |  | Jordan | 0 | 0 | 2037930 | 10359 | 0 | 2048289 | 7689760 | 26.64% |
|  |  | Palestine | 178920 | 1186829 | 3235998 | 8080 | 0 | 4609827 | 4904636 | 93.99% |
|  |  | Lebanon | 0 | 3202989 | 1626490 | 48639 | 17204 | 4895322 | 5053624 | 96.87% |
|  |  | Israel | 1437232 | 1615036 | 3963496 | 38779 | 28 | 7054571 | 7919528 | 89.08% |
|  |  | Turkey | 0 | 60537 | 8471848 | 1766489 | 6810 | 10305684 | 76690509 | 13.44% |
|  |  | Yemen | 0 | 296919 | 9719915 | 611808 | 0 | 10628642 | 25535086 | 41.62% |
|  |  | Syrian Arab Republic | 0 | 114603 | 10866037 | 328628 | 202 | 11309470 | 22264996 | 50.79% |
| **Europe** | Southern Europe | Croatia | 0 | 0 | 108 | 676 | 0 | 784 | 4255374 | 0.02% |
|  |  | Gibraltar | 0 | 20829 | 0 | 0 | 0 | 20829 | 29354 | 70.96% |
|  |  | Albania | 0 | 0 | 199210 | 73356 | 0 | 272566 | 3196981 | 8.53% |
|  |  | Malta | 0 | 319255 | 87123 | 0 | 0 | 406378 | 431239 | 94.23% |
|  |  | Greece | 0 | 9531 | 1211629 | 595588 | 24142 | 1840890 | 11125833 | 16.55% |
|  |  | Portugal | 172451 | 1663332 | 3702880 | 769437 | 299715 | 6607815 | 10610014 | 62.28% |
|  |  | Italy | 178310 | 777760 | 8237086 | 458176 | 0 | 9651332 | 61142221 | 15.79% |
|  |  | Spain | 510069 | 5714450 | 12213475 | 1956918 | 60804 | 20455716 | 47199069 | 43.34% |
|  | Central Europe | France | 0 | 0 | 1313569 | 464777 | 4348 | 1782694 | 64982894 | 2.74% |
| **Oceania** | Australia | New Zealand | 0 | 5356 | 1948153 | 515666 | 138610 | 2607785 | 4596396 | 56.74% |
|  |  | Australia | 7354260 | 2761434 | 2255207 | 515388 | 153348 | 13039637 | 23923101 | 54.51% |
|  | Melanesia | New Caledonia | 113226 | 54642 | 32108 | 221 | 0 | 200197 | 263147 | 76.08% |
|  |  | Vanuatu | 63495 | 96763 | 29729 | 584 | 0 | 190571 | 263888 | 72.22% |
|  |  | Solomon Islands | 68267 | 79505 | 196487 | 68034 | 140 | 412433 | 584482 | 70.56% |
|  |  | Fiji | 385593 | 246332 | 65098 | 1205 | 0 | 698228 | 892727 | 78.21% |
|  |  | Papua New Guinea | 318833 | 645159 | 3555160 | 1483553 | 23524 | 6026229 | 7631819 | 78.96% |
|  | Micronesia | Marshall Islands | 475 | 1040 | 171 | 0 | 0 | 1686 | 52993 | 3.18% |
|  |  | Kiribati | 0 | 3172 | 660 | 310 | 0 | 4142 | 105555 | 3.92% |
|  |  | Nauru | 5331 | 3650 | 0 | 0 | 0 | 8981 | 10122 | 88.73% |
|  |  | Palau | 0 | 4940 | 5677 | 1304 | 0 | 11921 | 21291 | 55.99% |
|  |  | Northern Mariana Islands | 26862 | 3664 | 0 | 0 | 0 | 30526 | 55070 | 55.43% |
|  |  | Micronesia, Federated States | 3741 | 28087 | 16521 | 591 | 0 | 48940 | 104460 | 46.85% |
|  |  | Guam | 96191 | 51597 | 551 | 30 | 0 | 148369 | 169885 | 87.33% |
|  | Polynesia | Tuvalu | 0 | 8 | 21 | 0 | 0 | 29 | 9916 | 0.29% |
|  |  | Niue | 0 | 595 | 411 | 0 | 0 | 1006 | 1610 | 62.48% |
|  |  | Wallis and Futuna Islands | 167 | 2423 | 895 | 0 | 0 | 3485 | 13153 | 26.50% |
|  |  | Cook Islands | 9968 | 590 | 0 | 360 | 0 | 10918 | 20833 | 52.41% |
|  |  | American Samoa | 19624 | 17859 | 9241 | 16 | 0 | 46740 | 55538 | 84.16% |
|  |  | Tonga | 58825 | 14020 | 0 | 0 | 0 | 72845 | 106379 | 68.48% |
|  |  | Samoa | 27012 | 76978 | 70795 | 490 | 0 | 175275 | 193228 | 90.71% |
|  |  | French Polynesia | 166291 | 48806 | 0 | 0 | 0 | 215097 | 282764 | 76.07% |
