# Supplementary material for: Global spatial assessment of Aedes aegypti and Culex quinquefasciatus: a scenario of Zika virus exposure
Source: Epidemiol Infect. 2018 Nov 26;147:e52. doi: 10.1017/S0950268818003102 (PMC6518585; doi:10.1017/S0950268818003102)
Supplement: Supplementary file 1 [file S0950268818003102sup001.zip › S0950268818003102sup001/Supplementary_data_table_5.docx]

**Supplementary table 5:** Comparison of people exposed to ZIKV due to *C. quinquefasciatus* and *A. aegypti* by country and continent. The column “% *Culex quinquefasciatus*” represents the percentage of people potentially exposed to ZIKV due to *C. quiquefasciatus*, while the column “% *Aedes aegypti*” shows the percentage due to *A. aegypti.* The column “% of difference” shows the difference of percentages of population exposed between both vectors (higher % - lower %), while the column “Preponderance” show how many times the vector has major percentage of population potentially affected than the other {higher % / lower %}. Violet color corresponds to countries where *A. aegypti* is preponderant (contributes with more potentially infected people), while yellow color appears in countries where *C. quinquefasciatus* has a major contribution to the risk. The empty boxes in blue color correspond to countries where only *A. aegypti* exists, while the empty boxes in orange color represent countries with only *C. quinquefasciatus*. The white boxes correspond to countries where both vectors are equally preponderant.

| **Region** | **Sub Region** | **Country** | **Population 2015** | **% *Culex quinquefasciatus*** | ***% Aedes aegypti*** | **% of difference** | **Preponderance** |
| --- | --- | --- | --- | --- | --- | --- | --- |
| **Africa** | Central Africa | Sao Tome and Principe | 202781 | 89.45% | 89.45% | 0.00% | 0.00 |
|  |  | Equatorial Guinea | 799372 | 89.71% | 98.02% | 8.32% | 0.08 |
|  |  | Gabon | 1725292 | 99.96% | 99.48% | 0.48% | 0.00 |
|  |  | Congo | 4671142 | 48.61% | 75.46% | 26.84% | 0.36 |
|  |  | Central African Republic | 4803082 | 91.85% | 91.98% | 0.13% | 0.00 |
|  |  | Chad | 13605625 | 28.73% | 48.76% | 20.03% | 0.41 |
|  |  | Angola | 22819926 | 37.52% | 99.71% | 62.19% | 0.62 |
|  |  | Cameroon | 23393129 | 91.50% | 95.95% | 4.46% | 0.05 |
|  |  | Democratic Republic of the Congo | 77266814 | 92.09% | 97.05% | 4.96% | 0.05 |
|  | East Africa | Seychelles | 93754 | 80.90% | 80.94% | 0.04% | 0.00 |
|  |  | Mayotte | 233993 | 80.70% | 80.66% | 0.04% | 0.00 |
|  |  | Comoros | 770058 | 89.20% | 88.13% | 1.07% | 0.01 |
|  |  | Reunion | 895099 | 94.30% | 82.99% | 11.31% | 0.14 |
|  |  | Djibouti | 899658 | 4.20% | 0.67% | 3.53% | 5.26 |
|  |  | Mauritius | 1253581 | 97.60% | 97.58% | 0.02% | 0.00 |
|  |  | Eritrea | 6737634 | 42.10% | 40.71% | 1.39% | 0.03 |
|  |  | Burundi | 10812619 | 91.20% | 90.48% | 0.72% | 0.01 |
|  |  | Somalia | 11122711 | 57.40% | 73.56% | 16.16% | 0.22 |
|  |  | Rwanda | 12428005 | 93.40% | 88.74% | 4.66% | 0.05 |
|  |  | Zimbabwe | 15046102 | 81.00% | 88.67% | 7.67% | 0.09 |
|  |  | Zambia | 15519604 | 19.60% | 80.41% | 60.81% | 0.76 |
|  |  | Malawi | 17308685 | 70.00% | 91.10% | 21.10% | 0.23 |
|  |  | Madagascar | 24235390 | 96.00% | 96.56% | 0.56% | 0.01 |
|  |  | Mozambique | 27121827 | 91.80% | 94.09% | 2.29% | 0.02 |
|  |  | Uganda | 40141262 | 94.70% | 93.60% | 1.10% | 0.01 |
|  |  | Kenya | 46748617 | 93.10% | 92.54% | 0.56% | 0.01 |
|  |  | United Republic of Tanzania | 52290796 | 78.70% | 89.11% | 10.41% | 0.12 |
|  |  | Ethiopia | 98942102 | 91.00% | 75.95% | 15.05% | 0.20 |
|  | North Africa | Western Sahara | 604298 | 0.10% | 0.00% |  |  |
|  |  | Libyan Arab Jamahiriya | 6317080 | 68.20% | 31.70% | 36.50% | 1.15 |
|  |  | Tunisia | 11235248 | 75.00% | 45.87% | 29.13% | 0.64 |
|  |  | Morocco | 34377511 | 89.90% | 98.89% | 8.99% | 0.09 |
|  |  | Sudan | 39613217 | 30.30% | 71.81% | 41.51% | 0.58 |
|  |  | Algeria | 40633464 | 58.70% | 23.05% | 35.65% | 1.55 |
|  |  | Egypt | 84705681 | 64.80% | 1.58% | 63.22% | 39.89 |
|  | Southern Africa | Swaziland | 1285519 | 82.52% | 81.86% | 0.67% | 0.01 |
|  |  | Botswana | 2056370 | 65.05% | 65.95% | 0.89% | 0.01 |
|  |  | Lesotho | 2120116 | 83.81% | 6.27% | 77.55% | 12.37 |
|  |  | Namibia | 2392370 | 23.91% | 27.45% | 3.55% | 0.13 |
|  |  | South Africa | 53491333 | 93.39% | 82.10% | 11.29% | 0.14 |
|  | West Africa | Saint Helena | 4124 | 67.00% | 67.02% | 0.02% | 0.00 |
|  |  | Niger | 19899120 | 0.00% | 1.23% |  |  |
|  |  | Cape Verde | 508315 | 28.70% | 79.64% | 50.94% | 0.64 |
|  |  | Mauritania | 4067564 | 0.00% | 27.65% |  |  |
|  |  | Guinea-Bissau | 1787793 | 29.30% | 90.52% | 61.22% | 0.68 |
|  |  | Gambia | 1970081 | 0.30% | 96.52% | 96.22% | 1.00 |
|  |  | Liberia | 4503439 | 88.80% | 88.88% | 0.08% | 0.00 |
|  |  | Sierra Leone | 6318575 | 96.60% | 96.57% | 0.03% | 0.00 |
|  |  | Togo | 7170797 | 99.90% | 99.89% | 0.01% | 0.00 |
|  |  | Benin | 10879828 | 91.00% | 98.49% | 7.49% | 0.08 |
|  |  | Guinea | 12347766 | 87.80% | 88.07% | 0.27% | 0.00 |
|  |  | Senegal | 14967446 | 2.70% | 84.58% | 81.88% | 0.97 |
|  |  | Mali | 16258587 | 21.40% | 80.21% | 58.81% | 0.73 |
|  |  | Burkina Faso | 17914625 | 31.50% | 91.40% | 59.90% | 0.66 |
|  |  | Cote d'Ivoire | 21295284 | 99.90% | 95.78% | 4.12% | 0.04 |
|  |  | Ghana | 26984328 | 98.10% | 99.71% | 1.61% | 0.02 |
|  |  | Nigeria | 183523432 | 64.60% | 78.65% | 14.05% | 0.18 |
| **America** | Caribbean | Montserrat | 5176 | 60.90% | 60.88% | 0.02% | 0.00 |
|  |  | Anguilla | 14614 | 52.60% | 52.59% | 0.01% | 0.00 |
|  |  | British Virgin Islands | 28800 | 87.80% | 87.84% | 0.04% | 0.00 |
|  |  | Turks and Caicos Islands | 34339 | 47.90% | 47.85% | 0.05% | 0.00 |
|  |  | Saint Kitts and Nevis | 55376 | 85.00% | 84.96% | 0.04% | 0.00 |
|  |  | Cayman Islands | 59967 | 45.70% | 45.68% | 0.02% | 0.00 |
|  |  | Dominica | 72680 | 89.60% | 89.60% | 0.00% | 0.00 |
|  |  | Saint Martin | 74853 | 36.60% | 36.64% | 0.04% | 0.00 |
|  |  | Antigua and Barbuda | 91822 | 87.70% | 89.56% | 1.86% | 0.02 |
|  |  | United States Virgin Islands | 103574 | 71.10% | 71.12% | 0.02% | 0.00 |
|  |  | Aruba | 103889 | 86.40% | 92.99% | 6.59% | 0.07 |
|  |  | Grenada | 106694 | 88.20% | 88.25% | 0.05% | 0.00 |
|  |  | Saint Vincent and the Grenadines | 109374 | 83.10% | 83.05% | 0.05% | 0.00 |
|  |  | Saint Lucia | 184937 | 85.90% | 85.87% | 0.03% | 0.00 |
|  |  | Netherlands Antilles | 227049 | 75.00% | 75.04% | 0.04% | 0.00 |
|  |  | Barbados | 287482 | 84.90% | 84.94% | 0.04% | 0.00 |
|  |  | Bahamas | 387549 | 65.00% | 65.05% | 0.05% | 0.00 |
|  |  | Martinique | 405688 | 92.20% | 92.24% | 0.04% | 0.00 |
|  |  | Guadeloupe | 470168 | 80.70% | 80.69% | 0.01% | 0.00 |
|  |  | Trinidad and Tobago | 1346697 | 90.40% | 90.50% | 0.10% | 0.00 |
|  |  | Jamaica | 2813276 | 94.90% | 94.89% | 0.01% | 0.00 |
|  |  | Puerto Rico | 3680058 | 95.30% | 95.28% | 0.02% | 0.00 |
|  |  | Haiti | 10603731 | 100.00% | 99.47% | 0.53% | 0.01 |
|  |  | Dominican Republic | 10652135 | 97.50% | 96.98% | 0.52% | 0.01 |
|  |  | Cuba | 11248783 | 97.10% | 97.09% | 0.01% | 0.00 |
|  | Central America | Bermuda | 65578 | 37.70% | 37.67% | 0.03% | 0.00 |
|  |  | Belize | 347598 | 98.00% | 98.03% | 0.03% | 0.00 |
|  |  | Panama | 3987866 | 90.50% | 90.30% | 0.20% | 0.00 |
|  |  | Costa Rica | 5001657 | 89.10% | 87.07% | 2.03% | 0.02 |
|  |  | Nicaragua | 6256510 | 98.80% | 98.81% | 0.01% | 0.00 |
|  |  | El Salvador | 6426002 | 97.50% | 97.53% | 0.03% | 0.00 |
|  |  | Honduras | 8423917 | 99.90% | 99.78% | 0.12% | 0.00 |
|  |  | Guatemala | 16255094 | 99.30% | 85.27% | 14.03% | 0.16 |
|  | Northern America | United States | 325127634 | 45.80% | 38.31% | 7.49% | 0.20 |
|  |  | Mexico | 125235587 | 96.80% | 77.15% | 19.65% | 0.25 |
|  | South America | French Guiana | 261729 | 73.40% | 73.44% | 0.04% | 0.00 |
|  |  | Suriname | 548456 | 91.90% | 91.90% | 0.00% | 0.00 |
|  |  | Guyana | 807611 | 73.80% | 73.84% | 0.04% | 0.00 |
|  |  | Uruguay | 3429997 | 88.30% | 88.33% | 0.03% | 0.00 |
|  |  | Paraguay | 7032942 | 99.50% | 99.79% | 0.29% | 0.00 |
|  |  | Bolivia | 11024522 | 84.70% | 47.67% | 37.03% | 0.78 |
|  |  | Ecuador | 16225691 | 89.20% | 62.00% | 27.20% | 0.44 |
|  |  | Chile | 17924062 | 83.30% | 10.12% | 73.18% | 7.23 |
|  |  | Peru | 31161167 | 38.00% | 16.72% | 21.28% | 1.27 |
|  |  | Venezuela | 31292702 | 97.20% | 99.89% | 2.69% | 0.03 |
|  |  | Argentina | 42154914 | 84.40% | 82.70% | 1.70% | 0.02 |
|  |  | Colombia | 49529208 | 99.90% | 74.02% | 25.88% | 0.35 |
|  |  | Brazil | 203657210 | 93.30% | 93.69% | 0.39% | 0.00 |
| **ASIA** | East Asia | Macau | 587606 | 22.40% | 22.42% | 0.02% | 0.00 |
|  |  | Korea, Republic of | 50293439 | 0.00% | 0.09% | 0.09% | 0.00 |
|  |  | Hong Kong | 7313557 | 77.00% | 77.01% | 0.01% | 0.00 |
|  |  | Taiwan | 8610384 | 36.74% | 98.13% | 61.39% | 0.63 |
|  |  | Japan | 126818019 | 7.60% | 4.26% | 3.34% | 0.78 |
|  |  | China | 1401586609 | 18.50% | 20.51% | 2.01% | 0.10 |
|  | South Asia | Maldives | 357981 | 0.33% | 0.33% | 0.00% | 0.00 |
|  |  | Bhutan | 776461 | 70.34% | 54.54% | 15.80% | 0.29 |
|  |  | Sri Lanka | 21611842 | 92.01% | 91.86% | 0.15% | 0.00 |
|  |  | Nepal | 28440629 | 99.71% | 98.66% | 1.05% | 0.01 |
|  |  | Afghanistan | 32006788 | 17.11% | 3.61% | 13.50% | 3.74 |
|  |  | Iran (Islamic Republic of) | 79476308 | 23.67% | 6.00% | 17.67% | 2.94 |
|  |  | Bangladesh | 160411249 | 93.83% | 93.83% | 0.00% | 0.00 |
|  |  | Pakistan | 188144040 | 93.90% | 71.45% | 22.45% | 0.31 |
|  |  | India | 1282390303 | 83.43% | 99.54% | 16.11% | 0.16 |
|  | Southeast Asia | christmas island | 2072 | 55.65% | 55.65% | 0.00% | 0.00 |
|  |  | Brunei Darussalam | 428539 | 91.92% | 91.92% | 0.00% | 0.00 |
|  |  | Timor-Leste | 1172668 | 99.82% | 97.26% | 2.56% | 0.03 |
|  |  | Singapore | 5618866 | 58.54% | 58.54% | 0.00% | 0.00 |
|  |  | Lao People's Democratic Republic | 7019652 | 99.68% | 99.65% | 0.03% | 0.00 |
|  |  | Cambodia | 15677059 | 96.21% | 99.40% | 3.19% | 0.03 |
|  |  | Malaysia | 30651176 | 96.61% | 96.60% | 0.01% | 0.00 |
|  |  | Burma | 54751920 | 85.74% | 90.41% | 4.67% | 0.05 |
|  |  | Thailand | 67400746 | 99.54% | 98.64% | 0.90% | 0.01 |
|  |  | Viet Nam | 93386630 | 96.85% | 96.72% | 0.13% | 0.00 |
|  |  | Philippines | 101802706 | 89.34% | 89.32% | 0.02% | 0.00 |
|  |  | Indonesia | 255708785 | 98.90% | 98.07% | 0.83% | 0.01 |
|  | Western Asia | Cyprus | 1164695 | 99.88% | 99.08% | 0.79% | 0.01 |
|  |  | Oman | 4490541 | 0.00% | 0.01% |  |  |
|  |  | United Arab Emirates | 9156963 | 0.00% | 0.10% |  |  |
|  |  | Tajikistan | 8481855 | 0.00% | 0.57% |  |  |
|  |  | Kuwait | 3583399 | 61.12% | 0.00% |  |  |
|  |  | Palestine | 4904636 | 100.04% | 93.99% | 6.05% | 0.06 |
|  |  | Lebanon | 5053624 | 99.17% | 96.87% | 2.30% | 0.02 |
|  |  | Jordan | 7689760 | 77.80% | 26.64% | 51.17% | 1.92 |
|  |  | Israel | 7919528 | 95.27% | 89.08% | 6.20% | 0.07 |
|  |  | Syrian Arab Republic | 22264996 | 84.58% | 50.79% | 33.79% | 0.67 |
|  |  | Yemen | 25535086 | 67.38% | 41.62% | 25.76% | 0.62 |
|  |  | Saudi Arabia | 29897741 | 2.82% | 0.40% | 2.42% | 6.09 |
|  |  | Iraq | 35766702 | 23.06% | 0.00% |  |  |
|  |  | Turkey | 76690509 | 45.66% | 13.44% | 32.23% | 2.40 |
| **Europe** | Southern Europe | Gibraltar | 29354 | 70.96% | 70.96% | 0.00% | 0.00 |
|  |  | San Marino | 31802 | 40.68% | 0.00% |  |  |
|  |  | Malta | 431239 | 94.24% | 94.23% | 0.00% | 0.00 |
|  |  | Montenegro | 621556 | 17.91% | 0.00% |  |  |
|  |  | The former Yugoslav Republic of Macedonia | 2082899 | 0.26% | 0.00% |  |  |
|  |  | Albania | 3196981 | 60.38% | 8.53% | 51.85% | 6.08 |
|  |  | Bosnia and Herzegovina | 3819684 | 8.13% | 0.00% |  |  |
|  |  | Croatia | 4255374 | 16.83% | 0.02% | 16.81% | 912.46 |
|  |  | Serbia | 9424030 | 0.97% | 0.00% |  |  |
|  |  | Portugal | 10610014 | 88.98% | 62.28% | 26.70% | 0.43 |
|  |  | Greece | 11125833 | 78.12% | 16.55% | 61.58% | 3.72 |
|  |  | Spain | 47199069 | 76.84% | 43.34% | 33.50% | 0.77 |
|  |  | Italy | 61142221 | 58.42% | 15.79% | 42.63% | 2.70 |
|  | Northern Europe | Guernsey | 62948 | 82.70% | 0.00% |  |  |
|  |  | Jersey | 100080 | 70.36% | 0.00% |  |  |
|  |  | United Kingdom | 63843856 | 41.00% | 0.00% |  |  |
|  | Central Europe | Monaco | 38320 | 43.01% | 0.00% |  |  |
|  |  | France | 64982894 | 62.02% | 2.74% | 59.28% | 21.64 |
|  | Western Europe | Bulgaria | 7112641 | 0.31% | 0.00% |  |  |
| **Oceania** | Australia | New Zealand | 4596396 | 75.53% | 56.74% | 18.79% | 0.33 |
|  |  | Australia | 23923101 | 79.42% | 54.51% | 24.91% | 0.46 |
|  | Melanesia | New Caledonia | 263147 | 76.07% | 76.08% | 0.00% | 0.00 |
|  |  | Vanuatu | 263888 | 72.21% | 72.22% | 0.00% | 0.00 |
|  |  | Solomon Islands | 584482 | 70.56% | 70.56% | 0.00% | 0.00 |
|  |  | Fiji | 892727 | 78.21% | 78.21% | 0.00% | 0.00 |
|  |  | Papua New Guinea | 7631819 | 92.31% | 78.96% | 13.35% | 0.17 |
|  | Micronesia | Nauru | 10122 | 52.67% | 88.73% | 36.06% | 0.41 |
|  |  | Palau | 21291 | 55.99% | 55.99% | 0.00% | 0.00 |
|  |  | Marshall Islands | 52993 | 1.22% | 3.18% | 1.96% | 0.62 |
|  |  | Northern Mariana Islands | 55070 | 55.43% | 55.43% | 0.00% | 0.00 |
|  |  | Micronesia, Federated States | 104460 | 46.85% | 46.85% | 0.00% | 0.00 |
|  |  | Kiribati | 105555 | 3.92% | 3.92% | 0.00% | 0.00 |
|  |  | Guam | 169885 | 87.32% | 87.33% | 0.02% | 0.00 |
|  | Polynesia | Tuvalu | 9916 | 0.29% | 0.29% | 0.00% | 0.00 |
|  |  | Niue | 1610 | 26.50% | 62.48% | 35.99% | 0.58 |
|  |  | Wallis and Futuna Islands | 13153 | 0.00% | 26.50% |  |  |
|  |  | Cook Islands | 20833 | 50.68% | 52.41% | 1.73% | 0.03 |
|  |  | American Samoa | 55538 | 84.16% | 84.16% | 0.00% | 0.00 |
|  |  | Tonga | 106379 | 68.48% | 68.48% | 0.00% | 0.00 |
|  |  | Samoa | 193228 | 90.71% | 90.71% | 0.00% | 0.00 |
|  |  | French Polynesia | 282764 | 76.07% | 76.07% | 0.00% | 0.00 |
