# Supplementary material for: Global spatial assessment of Aedes aegypti and Culex quinquefasciatus: a scenario of Zika virus exposure
Source: Epidemiol Infect. 2018 Nov 26;147:e52. doi: 10.1017/S0950268818003102 (PMC6518585; doi:10.1017/S0950268818003102)
Supplement: Supplementary file 1 [file S0950268818003102sup001.zip › S0950268818003102sup001/Supplementary_data_table__1_and_2_R2.docx]

**Supplementary Data, Table 1:** Sensitivity analysis of the predicted area per level of probability of presence to each vector expressed in km^2^/1000.

A) Total area per level of probability of presence to each scenario.

| ***A. aegypti*** | **Null** | **Low** | **Medium** | **High** |
| --- | --- | --- | --- | --- |
| SDM (95% CI) - SD | 277388.5 | 13515.4 | 25493.4 | 15363.4 |
| SDM (95% CI) | 277150.0 | 13527.8 | 25586.8 | 15494.9 |
| SDM (95% CI) + SD | 276898.0 | 13570.0 | 25663.5 | 15629.2 |
| ***C. quinquesaciatus*** | **Null** | **Low** | **Medium** | **High** |
| SDM (95% CI) - SD | 289469.4 | 30733.8 | 10687.8 | 869.7 |
| SDM (95% CI) | 288930.9 | 31321.4 | 10731.1 | 776.1 |
| SDM (95% CI) + SD | 288396.5 | 31893.9 | 10776.7 | 693.5 |

B) Difference between each scenario with the SDM (95% CI) for the levels of probability

| ***A. aegypti*** | **Null** | **Low** | **Medium** | **High** |
| --- | --- | --- | --- | --- |
| SDM (95% CI) - {SDM (95% CI) – SD} | 238.6 | -12.4 | -93.4 | -131.6 |
| SDM (95% CI) – {SDM (95% CI) + SD} | -252.0 | 42.2 | 76.7 | 134.3 |
| ***C. quinquesaciatus*** | **Null** | **Low** | **Medium** | **High** |
| SDM (95% CI) - {SDM (95% CI) – SD} | 538.5 | -587.6 | -43.3 | 93.6 |
| SDM (95% CI) – {SDM (95% CI) + SD} | -534.4 | 572.5 | 45.6 | -82.6 |

**Supplementary Data, Table 2:** Sensitivity analysis of the predicted interaction zones between *A. aegypti* and *C. quinquesaciatus* considering the two scenarios.

A) Total area per level of interaction to each scenario.

| **Scenario** | **Very low** | **Low** | **Medium** | **High** | **Very high** |
| --- | --- | --- | --- | --- | --- |
| SDM (95% CI) - SD | 5692.3 | 12407.6 | 13641.4 | 4892.5 | 352.6 |
| SDM (95% CI) | 5810.6 | 12705.0 | 13728.5 | 4960.3 | 301.4 |
| SDM (95% CI) + SD | 5937.1 | 13007.1 | 13810.7 | 5023.2 | 262.2 |

B) Difference between each scenario with the SDM (95% CI) for the levels of interaction.

| **Scenario** | **Very low** | **Low** | **Medium** | **High** | **Very high** |
| --- | --- | --- | --- | --- | --- |
| SDM (95% CI) - SD | 118.3 | 297.5 | 87.1 | 67.8 | -51.2 |
| SDM (95% CI) + SD | -126.4 | -302.0 | -82.3 | -62.9 | 39.3 |
