# Supplementary material for: Global spatial assessment of Aedes aegypti and Culex quinquefasciatus: a scenario of Zika virus exposure
Source: Epidemiol Infect. 2018 Nov 26;147:e52. doi: 10.1017/S0950268818003102 (PMC6518585; doi:10.1017/S0950268818003102)
Supplement: Supplementary file 1 [file S0950268818003102sup001.zip › S0950268818003102sup001/Supplementary_data_table__3.docx]

**Supplementary table 3:** Population potentially exposed to ZIKV based on the model of exposure to *Culex quinquefasciatus*, classified by risk level, country and continent. The column “% *Culex quinquefasciatus*” represents the percentage of the population potentially affected by country due to the contact with *C. quinquefasciatus*. Countries not listed present null risk of ZIKV due to this vector, according to our model.

| **Region** | **Sub Region** | **Country** | **Very high** | **High** | **Medium** | **Low** | **Very low** | **Total** | **Population 2015** | **% *Culex quinquefasciatus*** |
| --- | --- | --- | --- | --- | --- | --- | --- | --- | --- | --- |
| **Africa** | Central Africa | Sao Tome and Principe | 171292 | 10103 | 0 | 0 | 0 | 181395 | 202781 | 89.45% |
|  |  | Equatorial Guinea | 584609 | 109506 | 22970 | 0 | 0 | 717085 | 799372 | 89.71% |
|  |  | Gabon | 664573 | 377480 | 600245 | 82273 | 0 | 1724571 | 1725292 | 99.96% |
|  |  | Congo | 0 | 1097709 | 216508 | 675881 | 280751 | 2270849 | 4671142 | 48.61% |
|  |  | Central African Republic | 254492 | 1352459 | 2047459 | 700910 | 56170 | 4411490 | 4803082 | 91.85% |
|  |  | Chad | 0 | 13709 | 1521086 | 2173796 | 200373 | 3908964 | 13605625 | 28.73% |
|  |  | Angola | 902079 | 2865287 | 2368776 | 1724681 | 700216 | 8561039 | 22819926 | 37.52% |
|  |  | Cameroon | 5754446 | 7594175 | 6798498 | 1248540 | 8115 | 21403774 | 23393129 | 91.50% |
|  |  | Democratic Republic of the Congo | 11578057 | 27032298 | 21368439 | 6663301 | 4513521 | 71155616 | 77266814 | 92.09% |
|  | East Africa | Seychelles | 75888 | 0 | 0 | 0 | 0 | 75888 | 93754 | 80.94% |
|  |  | Mayotte | 188728 | 0 | 0 | 0 | 0 | 188728 | 233993 | 80.66% |
|  |  | Comoros | 686927 | 0 | 0 | 0 | 0 | 686927 | 770058 | 89.20% |
|  |  | Reunion | 779675 | 58923 | 5044 | 0 | 0 | 843642 | 895099 | 94.25% |
|  |  | Djibouti | 0 | 0 | 13577 | 24147 | 326 | 38050 | 899658 | 4.23% |
|  |  | Mauritius | 1143241 | 80009 | 0 | 0 | 0 | 1223250 | 1253581 | 97.58% |
|  |  | Eritrea | 1496278 | 354965 | 826583 | 159574 | 159 | 2837559 | 6737634 | 42.12% |
|  |  | Burundi | 9613082 | 242233 | 1596 | 0 | 0 | 9856911 | 10812619 | 91.16% |
|  |  | Somalia | 1648042 | 2225513 | 1856642 | 461661 | 197889 | 6389747 | 11122711 | 57.45% |
|  |  | Rwanda | 11550952 | 46513 | 4752 | 0 | 0 | 11602217 | 12428005 | 93.36% |
|  |  | Zimbabwe | 1301910 | 2335799 | 5092072 | 2472993 | 985750 | 12188524 | 15046102 | 81.01% |
|  |  | Zambia | 3117 | 454513 | 1249076 | 195590 | 1139741 | 3042037 | 15519604 | 19.60% |
|  |  | Malawi | 5362191 | 5815985 | 875637 | 66371 | 120 | 12120304 | 17308685 | 70.02% |
|  |  | Madagascar | 17616252 | 3095473 | 2049829 | 492985 | 0 | 23254539 | 24235390 | 95.95% |
|  |  | Mozambique | 15841530 | 5850230 | 2623054 | 506479 | 85654 | 24906947 | 27121827 | 91.83% |
|  |  | Uganda | 30876193 | 6608271 | 509441 | 5617 | 0 | 37999522 | 40141262 | 94.66% |
|  |  | Kenya | 37141480 | 3658097 | 2300272 | 429350 | 0 | 43529199 | 46748617 | 93.11% |
|  |  | United Republic of Tanzania | 21950605 | 13731474 | 5127876 | 326104 | 10769 | 41146828 | 52290796 | 78.69% |
|  |  | Ethiopia | 73100951 | 11348273 | 4946440 | 669423 | 3758 | 90068845 | 98942102 | 91.03% |
|  | North Africa | Western Sahara | 0 | 0 | 0 | 0 | 723 | 723 | 604298 | 0.12% |
|  |  | Libyan Arab Jamahiriya | 2378251 | 1081128 | 738226 | 110595 | 996 | 4309196 | 6317080 | 68.21% |
|  |  | Tunisia | 4506133 | 1755844 | 1638852 | 458417 | 72747 | 8431993 | 11235248 | 75.05% |
|  |  | Morocco | 16142266 | 6941579 | 5676131 | 1404503 | 372414 | 30536893 | 34377511 | 88.83% |
|  |  | Sudan | 400286 | 2207858 | 5759126 | 3074196 | 544500 | 11985966 | 39613217 | 30.26% |
|  |  | Algeria | 8883573 | 12065502 | 2435718 | 447637 | 21812 | 23854242 | 40633464 | 58.71% |
|  |  | Egypt | 7577921 | 17379920 | 24591966 | 5153096 | 228231 | 54931134 | 84705681 | 64.85% |
|  | Southern Africa | Swaziland | 558854 | 500269 | 1730 | 0 | 0 | 1060853 | 1285519 | 82.52% |
|  |  | Botswana | 40966 | 379659 | 312656 | 347180 | 257265 | 1337726 | 2056370 | 65.05% |
|  |  | Lesotho | 192790 | 751032 | 769875 | 63265 | 0 | 1776962 | 2120116 | 83.81% |
|  |  | Namibia | 141531 | 213382 | 84113 | 66306 | 66569 | 571901 | 2392370 | 23.91% |
|  |  | South Africa | 38612645 | 7129997 | 3550700 | 540941 | 121776 | 49956059 | 53491333 | 93.39% |
|  | West Africa | Saint Helena | 0 | 2450 | 314 | 0 | 0 | 2764 | 4124 | 67.02% |
|  |  | Cape Verde | 66808 | 57321 | 2986 | 14661 | 3903 | 145679 | 508315 | 28.66% |
|  |  | Guinea-Bissau | 86809 | 153978 | 282358 | 190 | 0 | 523335 | 1787793 | 29.27% |
|  |  | Gambia | 0 | 2967 | 3911 | 0 | 0 | 6878 | 1970081 | 0.35% |
|  |  | Liberia | 1865893 | 518381 | 1516606 | 98574 | 0 | 3999454 | 4503439 | 88.81% |
|  |  | Sierra Leone | 2783128 | 2129288 | 1157324 | 31919 | 0 | 6101659 | 6318575 | 96.57% |
|  |  | Togo | 3165213 | 1308777 | 2379633 | 309454 | 0 | 7163077 | 7170797 | 99.89% |
|  |  | Benin | 5833854 | 600121 | 3262481 | 202110 | 0 | 9898566 | 10879828 | 90.98% |
|  |  | Guinea | 6103295 | 2718760 | 1958117 | 60409 | 0 | 10840581 | 12347766 | 87.79% |
|  |  | Senegal | 0 | 0 | 405538 | 2618 | 0 | 408156 | 14967446 | 2.73% |
|  |  | Mali | 37375 | 534735 | 1590319 | 1228781 | 87162 | 3478372 | 16258587 | 21.39% |
|  |  | Burkina Faso | 0 | 382751 | 1846639 | 2302115 | 1117172 | 5648677 | 17914625 | 31.53% |
|  |  | Cote d'Ivoire | 8469323 | 7154856 | 5247915 | 400259 | 150 | 21272503 | 21295284 | 99.89% |
|  |  | Ghana | 15505146 | 5554360 | 3440334 | 1755778 | 226609 | 26482227 | 26984328 | 98.14% |
|  |  | Nigeria | 49844170 | 56029709 | 9123031 | 3567261 | 62459 | 118626630 | 183523432 | 64.64% |
| **America** | Caribbean | Montserrat | 1054 | 2097 | 0 | 0 | 0 | 3151 | 5176 | 60.88% |
|  |  | Anguilla | 7686 | 0 | 0 | 0 | 0 | 7686 | 14614 | 52.59% |
|  |  | British Virgin Islands | 25297 | 0 | 0 | 0 | 0 | 25297 | 28800 | 87.84% |
|  |  | Turks and Caicos Islands | 16433 | 0 | 0 | 0 | 0 | 16433 | 34339 | 47.86% |
|  |  | Saint Kitts and Nevis | 47050 | 0 | 0 | 0 | 0 | 47050 | 55376 | 84.96% |
|  |  | Cayman Islands | 27394 | 0 | 0 | 0 | 0 | 27394 | 59967 | 45.68% |
|  |  | Dominica | 43211 | 21914 | 0 | 0 | 0 | 65125 | 72680 | 89.61% |
|  |  | Saint Martin | 27423 | 0 | 0 | 0 | 0 | 27423 | 74853 | 36.64% |
|  |  | Antigua and Barbuda | 80541 | 0 | 0 | 0 | 0 | 80541 | 91822 | 87.71% |
|  |  | United States Virgin Islands | 73665 | 0 | 0 | 0 | 0 | 73665 | 103574 | 71.12% |
|  |  | Aruba | 89480 | 0 | 262 | 0 | 0 | 89742 | 103889 | 86.38% |
|  |  | Grenada | 94157 | 0 | 0 | 0 | 0 | 94157 | 106694 | 88.25% |
|  |  | Saint Vincent and the Grenadines | 82838 | 8001 | 0 | 0 | 0 | 90839 | 109374 | 83.05% |
|  |  | Saint Lucia | 158803 | 0 | 0 | 0 | 0 | 158803 | 184937 | 85.87% |
|  |  | Netherlands Antilles | 136089 | 30801 | 3488 | 0 | 0 | 170378 | 227049 | 75.04% |
|  |  | Barbados | 244198 | 0 | 0 | 0 | 0 | 244198 | 287482 | 84.94% |
|  |  | Bahamas | 248637 | 3462 | 0 | 0 | 0 | 252099 | 387549 | 65.05% |
|  |  | Martinique | 371086 | 3140 | 0 | 0 | 0 | 374226 | 405688 | 92.24% |
|  |  | Guadeloupe | 379402 | 0 | 0 | 0 | 0 | 379402 | 470168 | 80.69% |
|  |  | Trinidad and Tobago | 1134823 | 82820 | 0 | 0 | 0 | 1217643 | 1346697 | 90.42% |
|  |  | Jamaica | 2608511 | 60900 | 0 | 0 | 0 | 2669411 | 2813276 | 94.89% |
|  |  | Puerto Rico | 3184142 | 315567 | 6691 | 0 | 0 | 3506400 | 3680058 | 95.28% |
|  |  | Haiti | 10401968 | 206666 | 0 | 0 | 0 | 10608634 | 10603731 | 100.05% |
|  |  | Dominican Republic | 9468814 | 898652 | 16061 | 331 | 0 | 10383858 | 10652135 | 97.48% |
|  |  | Cuba | 9387244 | 1534489 | 0 | 0 | 0 | 10921733 | 11248783 | 97.09% |
|  | Central America | Bermuda | 24704 | 0 | 0 | 0 | 0 | 24704 | 65578 | 37.67% |
|  |  | Belize | 209811 | 111551 | 19408 | 0 | 0 | 340770 | 347598 | 98.04% |
|  |  | Panama | 2132987 | 1028252 | 397180 | 50459 | 0 | 3608878 | 3987866 | 90.50% |
|  |  | Costa Rica | 3209567 | 1055614 | 187770 | 3007 | 0 | 4455958 | 5001657 | 89.09% |
|  |  | Nicaragua | 3959750 | 2178407 | 43776 | 0 | 0 | 6181933 | 6256510 | 98.81% |
|  |  | El Salvador | 5938683 | 316844 | 12003 | 0 | 0 | 6267530 | 6426002 | 97.53% |
|  |  | Honduras | 6024950 | 2130853 | 257952 | 331 | 0 | 8414086 | 8423917 | 99.88% |
|  |  | Guatemala | 11267250 | 4615813 | 266088 | 0 | 0 | 16149151 | 16255094 | 99.35% |
|  | North America | United States | 81613260 | 27770878 | 22176634 | 13568108 | 3818214 | 148947094 | 325127634 | 45.81% |
|  |  | Mexico | 107574516 | 9328462 | 2542794 | 1743130 | 45437 | 121234339 | 125235587 | 96.81% |
|  | South America | French Guiana | 118414 | 68987 | 4807 | 0 | 0 | 192208 | 261729 | 73.44% |
|  |  | Suriname | 418719 | 37114 | 48215 | 0 | 0 | 504048 | 548456 | 91.90% |
|  |  | Guyana | 496365 | 78545 | 20051 | 1360 | 13 | 596334 | 807611 | 73.84% |
|  |  | Uruguay | 2769100 | 243858 | 16733 | 0 | 0 | 3029691 | 3429997 | 88.33% |
|  |  | Paraguay | 6245822 | 718804 | 33653 | 0 | 0 | 6998279 | 7032942 | 99.51% |
|  |  | Bolivia | 5068597 | 2716228 | 1373087 | 147685 | 36069 | 9341666 | 11024522 | 84.74% |
|  |  | Ecuador | 8481126 | 4565008 | 1114628 | 274234 | 36982 | 14471978 | 16225691 | 89.19% |
|  |  | Chile | 9994180 | 2603449 | 1595961 | 558072 | 185555 | 14937217 | 17924062 | 83.34% |
|  |  | Peru | 4276911 | 4042721 | 2673266 | 781881 | 81396 | 11856175 | 31161167 | 38.05% |
|  |  | Venezuela | 15424882 | 9805622 | 4364577 | 816204 | 0 | 30411285 | 31292702 | 97.18% |
|  |  | Argentina | 27852829 | 5572118 | 1808868 | 273455 | 54249 | 35561519 | 42154914 | 84.36% |
|  |  | Colombia | 17519341 | 21727405 | 9142212 | 1016218 | 59797 | 49464973 | 49529208 | 99.87% |
|  |  | Brazil | 135829794 | 33629258 | 16925397 | 3345217 | 369869 | 190099535 | 203657210 | 93.34% |
| **ASIA** | East Asia | Macau | 131728 | 0 | 0 | 0 | 0 | 131728 | 587606 | 22.42% |
|  |  | Hong Kong | 4993146 | 638688 | 0 | 0 | 0 | 5631834 | 7313557 | 77.01% |
|  |  | Taiwan | 8247570 | 240880 | 68969 | 32211 | 0 | 8589630 | 23381038 | 36.74% |
|  |  | Japan | 1480394 | 2602049 | 4316067 | 1199193 | 43852 | 9641555 | 126818019 | 7.60% |
|  |  | China | 161435918 | 53048209 | 31605279 | 11629970 | 933125 | 258652501 | 1401586609 | 18.45% |
|  | South Asia | Maldives | 1184 | 0 | 0 | 0 | 0 | 1184 | 357981 | 0.33% |
|  |  | Bhutan | 143626 | 221256 | 157496 | 23818 | 0 | 546196 | 776461 | 70.34% |
|  |  | Sri Lanka | 17567123 | 2141253 | 174920 | 2394 | 0 | 19885690 | 21611842 | 92.01% |
|  |  | Nepal | 24197579 | 3509034 | 638416 | 12441 | 0 | 28357470 | 28440629 | 99.71% |
|  |  | Afghanistan | 0 | 1663472 | 1213523 | 2435596 | 164032 | 5476623 | 32006788 | 17.11% |
|  |  | Iran (Islamic Republic of) | 5096279 | 3753642 | 4754741 | 3804181 | 1402345 | 18811188 | 79476308 | 23.67% |
|  |  | Bangladesh | 150228180 | 283320 | 0 | 0 | 0 | 150511500 | 160411249 | 93.83% |
|  |  | Pakistan | 126687447 | 42241646 | 4752955 | 2152876 | 823312 | 176658236 | 188144040 | 93.90% |
|  |  | India | 762275723 | 270029921 | 33563043 | 4012829 | 22895 | 1069904411 | 1282390303 | 83.43% |
|  | Southeast Asia | Christmas Island | 0 | 1153 | 0 | 0 | 0 | 1153 | 2072 | 55.65% |
|  |  | Brunei Darussalam | 348126 | 31960 | 11356 | 2482 | 0 | 393924 | 428539 | 91.92% |
|  |  | Timor-Leste | 755841 | 405136 | 9579 | 0 | 0 | 1170556 | 1172668 | 99.82% |
|  |  | Singapore | 3001080 | 288062 | 0 | 0 | 0 | 3289142 | 5618866 | 58.54% |
|  |  | Lao People's Democratic Republic | 1773641 | 4407401 | 815156 | 754 | 0 | 6996952 | 7019652 | 99.68% |
|  |  | Cambodia | 11923547 | 2907106 | 251773 | 753 | 0 | 15083179 | 15677059 | 96.21% |
|  |  | Malaysia | 12624042 | 13223296 | 3469237 | 296312 | 0 | 29612887 | 30651176 | 96.61% |
|  |  | Burma | 20156575 | 20383205 | 6032381 | 371474 | 0 | 46943635 | 54751920 | 85.74% |
|  |  | Thailand | 44168971 | 18246640 | 4662954 | 14259 | 0 | 67092824 | 67400746 | 99.54% |
|  |  | Viet Nam | 82020908 | 7998743 | 426451 | 0 | 0 | 90446102 | 93386630 | 96.85% |
|  |  | Philippines | 75567886 | 12496872 | 2816823 | 65595 | 0 | 90947176 | 101802706 | 89.34% |
|  |  | Indonesia | 149828132 | 78992597 | 22904893 | 1161251 | 20075 | 252906948 | 255708785 | 98.90% |
|  | Western Asia | Cyprus | 1037175 | 92367 | 28695 | 5035 | 0 | 1163272 | 1164695 | 99.88% |
|  |  | Kuwait | 0 | 722530 | 1361321 | 106315 | 0 | 2190166 | 3583399 | 61.12% |
|  |  | Palestine | 4836080 | 68910 | 0 | 1611 | 0 | 4906601 | 4904636 | 100.04% |
|  |  | Lebanon | 3836916 | 1055787 | 114627 | 4413 | 0 | 5011743 | 5053624 | 99.17% |
|  |  | Jordan | 2076542 | 3706206 | 158689 | 40876 | 608 | 5982921 | 7689760 | 77.80% |
|  |  | Israel | 7344410 | 136835 | 59207 | 4655 | 83 | 7545190 | 7919528 | 95.27% |
|  |  | Syrian Arab Republic | 2329844 | 13047540 | 2391860 | 832997 | 229867 | 18832108 | 22264996 | 84.58% |
|  |  | Yemen | 11747365 | 4240644 | 1028178 | 189274 | 772 | 17206233 | 25535086 | 67.38% |
|  |  | Saudi Arabia | 0 | 4474 | 240181 | 209267 | 387805 | 841727 | 29897741 | 2.82% |
|  |  | Iraq | 2903067 | 985157 | 2049093 | 2190072 | 119931 | 8247320 | 35766702 | 23.06% |
|  |  | Turkey | 5499243 | 6592384 | 4504410 | 17821249 | 602654 | 35019940 | 76690509 | 45.66% |
| **Europe** | Southern Europe | Gibraltar | 20829 | 0 | 0 | 0 | 0 | 20829 | 29354 | 70.96% |
|  |  | San Marino | 0 | 0 | 12938 | 0 | 0 | 12938 | 31802 | 40.68% |
|  |  | Malta | 406381 | 0 | 0 | 0 | 0 | 406381 | 431239 | 94.24% |
|  |  | Montenegro | 0 | 3681 | 78198 | 29439 | 0 | 111318 | 621556 | 17.91% |
|  |  | The former Yugoslav Republic of Macedonia | 0 | 0 | 0 | 0 | 5414 | 5414 | 2082899 | 0.26% |
|  |  | Albania | 544665 | 1046241 | 322236 | 17180 | 0 | 1930322 | 3196981 | 60.38% |
|  |  | Bosnia and Herzegovina | 8703 | 65225 | 19411 | 145686 | 71392 | 310417 | 3819684 | 8.13% |
|  |  | Croatia | 35695 | 260610 | 128416 | 186424 | 105004 | 716149 | 4255374 | 16.83% |
|  |  | Serbia | 0 | 0 | 0 | 59102 | 31956 | 91058 | 9424030 | 0.97% |
|  |  | Portugal | 5554921 | 2714667 | 849714 | 291790 | 29869 | 9440961 | 10610014 | 88.98% |
|  |  | Greece | 1899826 | 3953675 | 1706518 | 795708 | 335925 | 8691652 | 11125833 | 78.12% |
|  |  | Spain | 16382803 | 11380792 | 5796383 | 2215528 | 491578 | 36267084 | 47199069 | 76.84% |
|  |  | Italy | 13212845 | 9710091 | 8144603 | 4443444 | 207857 | 35718840 | 61142221 | 58.42% |
|  |  | Guernsey | 30898 | 21163 | 0 | 0 | 0 | 52061 | 62948 | 82.70% |
|  | Northern Europe | Jersey | 23400 | 47014 | 0 | 0 | 0 | 70414 | 100080 | 70.36% |
|  |  | United Kingdom | 0 | 2010668 | 2759392 | 18861416 | 2542701 | 26174177 | 63843856 | 41.00% |
|  | Central Europe | Monaco | 0 | 15641 | 839 | 0 | 0 | 16480 | 38320 | 43.01% |
|  |  | France | 6560297 | 7195466 | 8117205 | 16651324 | 1778307 | 40302599 | 64982894 | 62.02% |
|  | Western Europe | Bulgaria | 0 | 0 | 0 | 2408 | 19383 | 21791 | 7112641 | 0.31% |
| **Oceania** | Australia | New Zealand | 2375780 | 667871 | 252916 | 133911 | 40999 | 3471477 | 4596396 | 75.53% |
|  |  | Australia | 15229465 | 2051195 | 985118 | 509587 | 223858 | 18999223 | 23923101 | 79.42% |
|  | Melanesia | New Caledonia | 144347 | 33550 | 22291 | 0 | 0 | 200188 | 263147 | 76.07% |
|  |  | Vanuatu | 141411 | 45788 | 3365 | 0 | 0 | 190564 | 263888 | 72.21% |
|  |  | Solomon Islands | 132027 | 194872 | 78247 | 7261 | 0 | 412407 | 584482 | 70.56% |
|  |  | Fiji | 618879 | 68486 | 10875 | 0 | 0 | 698240 | 892727 | 78.21% |
|  |  | Papua New Guinea | 1665594 | 3452810 | 1891453 | 35282 | 0 | 7045139 | 7631819 | 92.31% |
|  | Micronesia | Nauru | 5331 | 0 | 0 | 0 | 0 | 5331 | 10122 | 52.67% |
|  |  | Palau | 5608 | 5009 | 1304 | 0 | 0 | 11921 | 21291 | 55.99% |
|  |  | Marshall Islands | 563 | 83 | 0 | 0 | 0 | 646 | 52993 | 1.22% |
|  |  | Northern Mariana Islands | 27362 | 3163 | 0 | 0 | 0 | 30525 | 55070 | 55.43% |
|  |  | Micronesia, Federated States | 24012 | 17091 | 7835 | 0 | 0 | 48938 | 104460 | 46.85% |
|  |  | Kiribati | 3172 | 660 | 310 | 0 | 0 | 4142 | 105555 | 3.92% |
|  |  | Guam | 122965 | 24823 | 551 | 0 | 0 | 148339 | 169885 | 87.32% |
|  | Polynesia | Tuvalu | 8 | 21 | 0 | 0 | 0 | 29 | 9916 | 0.29% |
|  |  | Wallis and Futuna Islands | 2590 | 895 | 0 | 0 | 0 | 3485 | 13153 | 26.50% |
|  |  | Cook Islands | 10558 | 0 | 0 | 0 | 0 | 10558 | 20833 | 50.68% |
|  |  | American Samoa | 37483 | 9241 | 16 | 0 | 0 | 46740 | 55538 | 84.16% |
|  |  | Tonga | 71997 | 848 | 0 | 0 | 0 | 72845 | 106379 | 68.48% |
|  |  | Samoa | 159785 | 15490 | 0 | 0 | 0 | 175275 | 193228 | 90.71% |
|  |  | French Polynesia | 193367 | 21726 | 0 | 0 | 0 | 215093 | 282764 | 76.07% |
